# Unlocking hepatocellular carcinoma aggression: STAMBPL1-mediated TRAF2 deubiquitination activates WNT/PI3K/NF-kb signaling pathway

**DOI:** 10.1186/s13062-024-00460-7

**Published:** 2024-02-28

**Authors:** Zhihuai Wang, Yinjie Zhang, Yuhang Shen, Haiyang Zhou, Yuan Gao, Chunfu Zhu, Xihu Qin

**Affiliations:** 1 Nanjing Medical University, Nanjing, 211166 China; 2 Department of General Surgery, The Affiliated Changzhou No. 2 People’s Hospital of Nanjing Medical University, Changzhou, 213000 China

## Abstract

STAM Binding Protein Like 1 (STAMBPL1), functions as a deubiquitinase (DUB) and plays a significant role in various types of cancers. However, its effect as a DUB participating in the HCC tumorigenesis and progression still unknown. In the study, the upregulation and strong prognosis value of STAMBPL1 were identified in HCC patients. Functionally, STAMBPL1 significantly promoted HCC cells proliferation and metastasis, and it interacts with TRAF2 and stabilize it via the deubiquitination at the K63 residue. The TRAF2 upregulation stabilized by STAMBPL1 overexpression transfers of P65 protein into the nucleus and activates the WNT/PI3K/ NF-kb signaling pathway. The 251–436 sites of STAMBPL1 particularly interact with the 294–496 sites of TRAF2, thereby exerting the function of DUB and removing the ubiquitin molecules attached to TRAF2. Our research unveiled a new function of STAMBPL1 in mediating TRAF2 deubiquitination and stabilization, thereby activating the WNT/PI3K/NF-kb signaling pathway, suggesting its potential as a novel biomarker and therapeutic target for HCC.

**Supplementary Information:**

The online version contains supplementary material available at 10.1186/s13062-024-00460-7.

## Introduction

Hepatocellular carcinoma (HCC) is one of the most lethal malignancies around the world. Most HCC patients were diagnosed in later period because of lacking reliable diagnosed biomarkers [1]. Although there are a few of therapeutic methods such as surgery, targeted therapy, immunotherapy, and radiotherapy, the overall survival (OS) and progression free survival (PFS) rates of HCC patients [2]. Thus, it is necessary to explore novel diagnosed biomarkers and therapeutic targets to improve the diagnosis and treatment of HCC patients.

Deubiquitination is a process that removes ubiquitin from proteins, reversing the ubiquitination process that typically targets proteins for degradation by the proteasome [3]. This process is mediated by deubiquitinating enzymes (DUBs), which can have various roles in cellular processes, including cell cycle regulation [4], DNA repair [5], and signal transduction [6, 7]. Deubiquitination has been shown to play a significant role in the progression and treatment of cancer progression [8]. Specifically, in prostate cancer, the deubiquitinase USP22 has been found to be overexpressed with disease progression and is associated with poor prognosis, making it a potential therapeutic target [9]. USP11 mediated the deubiquitination of LSH and inhibited ferroptosis in colorectal cancer through epigenetic activation of CYP24A1, suggesting a role in cancer cell survival [10]. Several research have highlighted the importance of DUBs in HCC. For instance, USP5 has been identified as a DUB for LSH, and its activity is linked to the ferroptosis suppression, thereby facilitating the tumorigenesis of HCC [11]. USP39 promotes HCC cell proliferation and migration by deubiquitinating β-catenin, activating WNT/β-catenin signaling pathway [12]. These findings underscore the complex role of deubiquitination in HCC, with various DUBs contributing to the progression of the disease by stabilizing oncoproteins or inhibiting cell death pathways. As such, DUBs offer promising targets for the advancement of novel therapeutic approaches in the treatment of HCC.

STAMBPL1 (STAM Binding Protein Like 1) is a protein that functions as a deubiquitinase with 50 kDa Molecular weight, which is an enzyme that removes ubiquitin from proteins, thereby regulating their degradation, localization, or activity. STAMBPL1 has been implicated in diverse cancer types, notably colorectal cancer, as reported in the literature [13], gastric cancer [14] and lung cancer [15]. Especially its knockdown reduced cell viability, suppressed proliferation, invasion, and migration, and suppressed tumor growth of CRC [13]. STAMBPL1 has been recognized for its functional involvement in driving the epithelial–mesenchymal transition (EMT) process in malignant carcinoma, contributing to metastasis induction [15]. As of now, there is a lack of literature documenting the involvement of STAMBPL1 in HCC.

In our research, we sought to explore the biological roles and mechanisms of STAMBPL1 in influencing the aggressive progression of HCC, and to establish the viability of STAMBPL1 as a potential therapeutic target.

## Results

### STAMBPL1 has a higher expression in HCC tissues

**Fig. 1:**
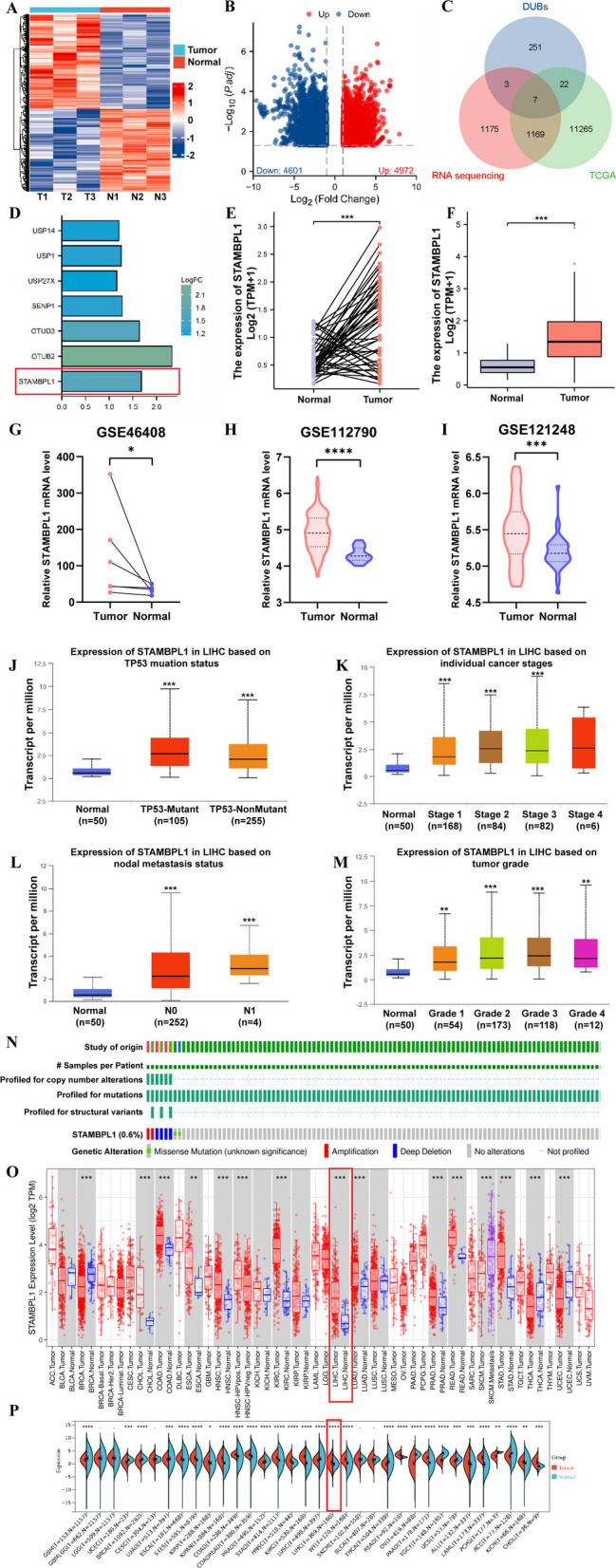
**A** Heatmap represents the results of RNA-seq on 3 pairs of HCC tissue and the corresponding adjacent non-cancerous tissue. **B** The volcano plot displays that 4972 genes are upregulated, and 4602 genes are downregulated in the RNA-seq results. **C** By intersecting the differential expressed genes (DEGs) from TCGA, the ubiquitinase list (DUBs), and DEGs of RNA-seq, we ultimately obtained 7 genes. **D** The bar chart displays the fold change of 7 genes (USP14, USP1, USP27X, SENP1, OTUD3, OTUB2, STAMBPL1) expression in HCC compared to the non-cancerous tissue. **E** By utilizing paired t-test analysis on gene expression data and corresponding patient information from the TCGA database, the high expression of STAMBPL1 was identified. **F** By utilizing independent samples t-test analysis on gene expression data and corresponding patient information from the TCGA database, the high expression of STAMBPL1 was identified. **G** Through the application of paired t-test analysis on gene expression data alongside corresponding patient information from the GSE46408 dataset, the high expression of STAMBPL1 was identified. **H** By utilizing independent samples t-test analysis on gene expression data and corresponding patient information from the GSE112790 dataset, the high expression of STAMBPL1 was identified. **I** By utilizing independent samples t-test analysis on gene expression data and corresponding patient information from the GSE121248 dataset, the high expression of STAMBPL1 was identified. **J** The expression of STAMBPL1 is significantly correlated with TP53 mutations. **K** The expression of STAMBPL1 is significantly correlated with the pathological stage of HCC patients. **L** The expression of STAMBPL1 is significantly correlated with the presence or absence of lymph node metastasis. **M** The STAMBPL1 expression shows a significant correlation with the pathological grade of patients with HCC. **N** The cBioPortal database indicates that mutations in STAMBPL1 predominantly include amplifications, deletions, and missense mutations. **O** The TIMER online database confirmed that STAMBPL1 is significantly overexpressed in pan-cancer, including in HCC. **P** Further confirmation was made through the use of the TCGA database that STAMBPL1 is significantly overexpressed in pan-cancer, including in HCC

**Fig. 2:**
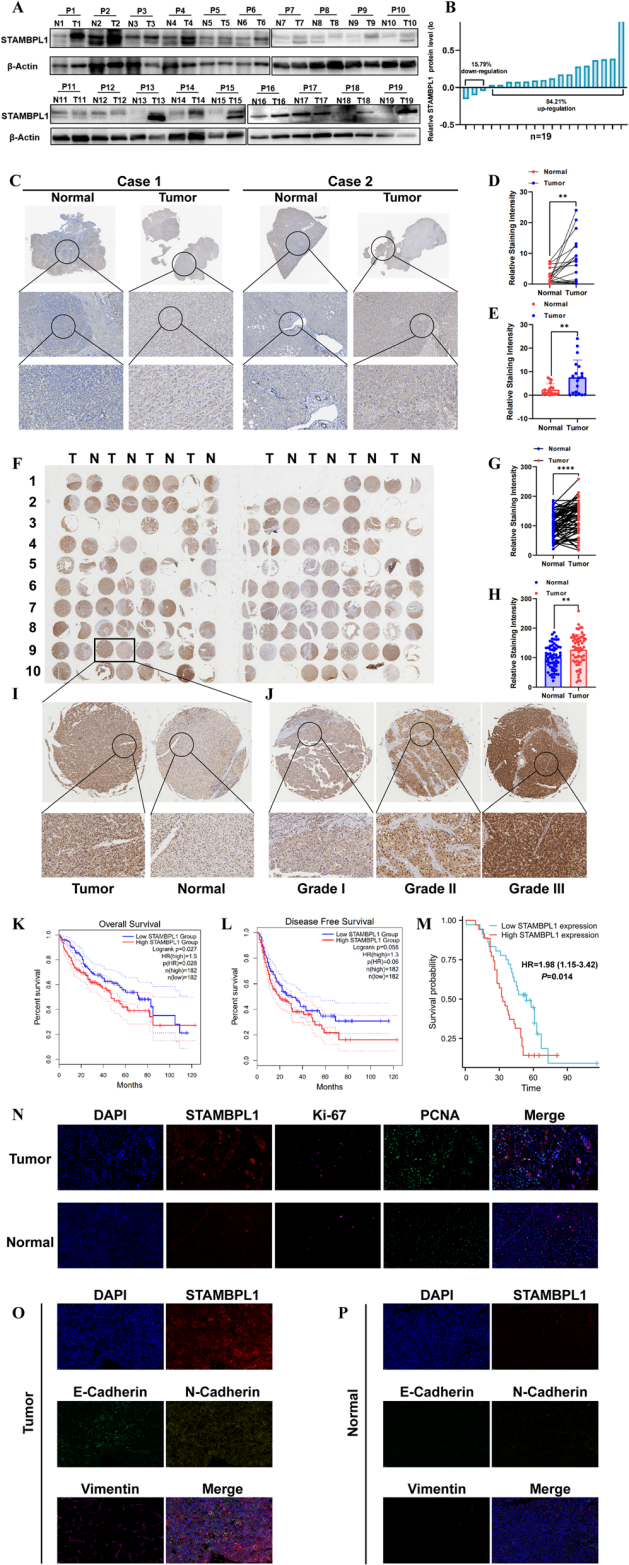
**A, B** Through Western blot (WB) analysis in 19 pairs of HCC samples, it was found that STAMBPL1 is primarily significantly overexpressed in HCC. **C–E** Detection through immunohistochemistry (IHC) in 19 pairs of HCC samples revealed a significantly high expression of STAMBPL1, primarily in HCC. **F–I** Further analysis using tissue microarray (90 pairs of liver cancer and adjacent tissues) revealed that STAMBPL1 is significantly highly expressed, particularly in HCC. **J** Further confirmation through tissue microarray analysis (90 pairs of HCC and adjacent tissues) validated a significant positive correlation between the expression level of STAMBPL1 and the pathological grade of HCC. **K** Using the GEPIA online database, it was found that the overall survival (OS) is shorter in the high STAMBPL1 expression group compared to the low STAMBPL1 expression group. **L** Using the GEPIA online database, it was found that the disease-free survival (DFS) is shorter in the high STAMBPL1 expression group compared to the low STAMBPL1 expression group. **M** By combining tissue microarray data with the corresponding patient survival data, it was observed that the high STAMBPL1 expression exhibits a reduced overall survival duration when contrasted with the low expression group. **N** The Immunofluorescence staining assay shows that STAMBPL1, along with the common tumor antigens Ki-67 and PCNA, are highly expressed in tumor tissues. **O–P** The Immunofluorescence assay showed that STAMBPL1, E-Cadherin, Vimentin, and N-Cadherin were all highly expressed in tumor tissues, and their expression trends were positively correlated

The results of RNA-sequencing based on 3 pairs of HCC and paracancerous tissues were showed by heatmap (Fig. 1A), the DEGs including 4972 upregulated genes and 4602 downregulated genes are presented in the volcano plot (Fig. 1B). The intersection was carried out among DEGs, deubiquitinating enzymes (DUBs) gene list and DEGs originated from TCGA database (Fig. 1C). Seven key genes were screened out which including USP14, USP1, USP27X, SENP1, OTUD3, OTUB2 and STAMBPL1 (Fig. 1D). There is no related research on the effect of STAMBPL1 in HCC, so we focus on the concrete carcinogenic mechanism of STAMBPL1 in HCC. The LIHC-TCGA dataset verified that STAMBPL1 is highly expressed in HCC by using paired t test (Fig. 1E) and unpaired t test (Fig. 1F). Besides, the expression data of STAMBPL1 in GSE46408 dataset, GSE112790 dataset and GSE121248 dataset also demonstrated that STAMBPL1 have a higher expression in HCC tissues than in paracancerous tissues (Fig. 1G–I). The expression of STAMBPL1 in HCC tissues with TP53-Mutant or TP53-NonMutant all showed a higher expression than normal liver tissues, and the STAMBPL1 is highly expressed in TP53 mutant HCC specimen compared with TP53-nonmutant type (Fig. 1J). Through the analysis of TCGA expression symbol and clinicopathological information, it was found that the expression of STAMBPL1 increased with the variation of tumor stage (Fig. 1K) and grade (Fig. 1M) in HCC. The STAMBPL1 expression is elevated in N1 stage compared to N0 stage of HCC patients (Fig. 1L). By searching on the online CbioPortal website, the result indicated that 0.6% mutation occurred in the profile of structural variants, and mutations in STAMBPL1 predominantly include amplifications, deletions, and missense mutations (Fig. 1N). The TIMER online tumor Cancer research database and TCGA database indicated that STAMBPL1 is generally highly expressed in a majority of tumor types including HCC (Fig. 1O–P). The Integrative Molecular Database of Hepatocellular Carcinoma database (HCCDB) database further confirmed the upregulation of STAMBPL1 in HCC tissues compared with paracancerous tissues (Supplementary Fig. S1A–C). The single cell sequencing data and the Spatial transcriptome data showed that the high STAMBPL1 expression is mainly located in tumor cells (Supplementary Fig. S1D-E). The results of WB based on 19 pairs of HCC and paracancerous tissues showed that STAMBPL1 expressed higher in most HCC tissues (Fig. 2A, B). The typical IHC images of the 19 pairs of HCC and paracancerous tissues further confirmed the high expression of STAMBPL1 in HCC (Fig. 2C), the significance difference was verified by using paired t test and unpaired t test (Fig. 2D–E). In order to deeply validate the upregulation of STAMBPL1, the HCC TMA was used and upregulation of STAMBPL1 was verified again (Fig. 2F–I). The patients with later pathological grade always had a higher expression of STAMBPL1 (Fig. 2J). According to the mRNA and clinical data from TCGA data, The K-M curves showed that patients with higher expression of STAMBPL1 always have worse OS and DFS rates and prognosis (Fig. 2K–L). By combing the IHC score of HCC-TMA and corresponding clinicopathological information, the K-M curve was drawn and the curve indicated the patients with high STAMBPL1 expression had worse prognosis and shorter survival time (Fig. 2M). Analysis of tissue immunofluorescence unveiled a substantial correlation between STAMBPL1 expression and the presence of PCNA and Ki67 in both hepatocellular carcinoma (HCC) tissue and adjacent non-cancerous tissues (Fig. 2N). At the same time, the expression of STAMBPL1 shows a significant positive correlation with the expression of E-Cadherin, N-Cadherin, and Vimentin. All of them are significantly overexpressed in tumor tissue (Fig. 2O–P).

### STAMBPL1 influences the proliferation and metastasis of HCC in vitro

**Fig. 3:**
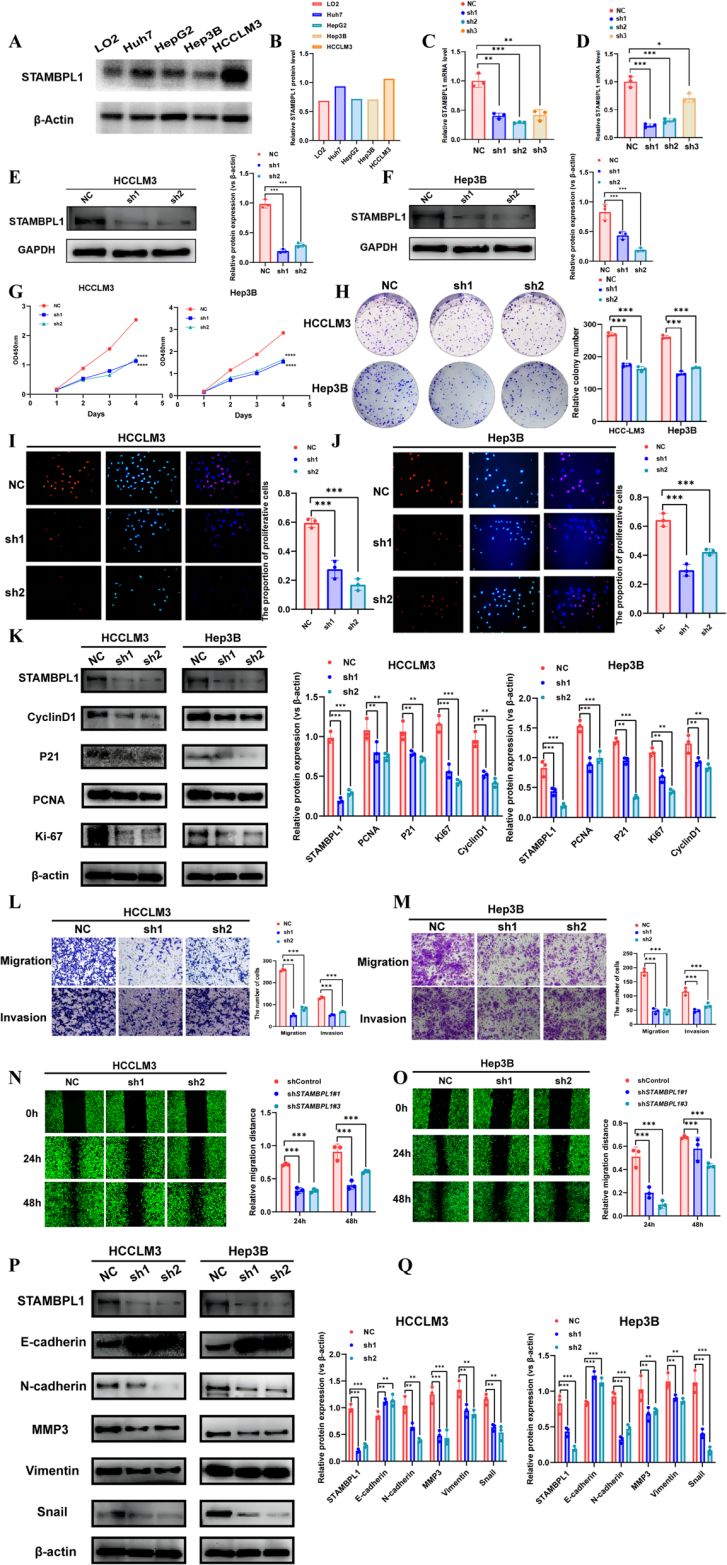
**A, B** Analyzing the expression levels of STAMBPL1 in five different types of HCC cells through Western blot analysis. **C** Validating the knockdown efficiency of shRNA targeting STAMBPL1 in HCCLM3 cells using qRT-PCR. **D** Validating the knockdown efficiency of shRNA targeting STAMBPL1 in Hep3B cells using qRT-PCR. **E** Western blot validation of the knockdown efficiency of shRNA targeting STAMBPL1 in HCCLM3 cell. **F** Western blot validation of the knockdown efficiency of shRNA targeting STAMBPL1 in Hep3B cell. **G** CCK-8 experiment to assess the proliferation efficiency between the sh-control group and the sh-STAMBPL1 group in HCCLM3 and Hep3B cell. **H** The colony formation assay to examine the proliferation efficiency of the sh-control group and the sh-STAMBPL1 group HCCLM3 and Hep3B cell. **I** The EdU assay to assess the proliferation efficiency between sh-control group and the sh-STAMBPL1 group in HCCLM3 cell. **J** The EdU assay to assess the proliferation efficiency between sh-control group and the sh-STAMBPL1 group in Hep3B cell. **K** Performing Western blot analysis to identify the Cyclin D1, P21, PCNA, and Ki-67 expression in both the sh-control and sh-STAMBPL1 groups of HCCLM3 and Hep3B cells. **L** The transwell assay to evaluate the migration and invasion efficiency between the sh-control and sh-STAMBPL1 group of HCCLM3 cell. **M** The transwell assay to evaluate the migration and invasion efficiency between the sh-control and sh-STAMBPL1 group of Hep3B cell. **N** The cell scratch assay to assess the migration efficiency of the sh-control and sh-STAMBPL1 group in HCCLM3 cell. **O** The cell scratch assay to assess the migration efficiency of the sh-control and sh-STAMBPL1 group in Hep3B cell. **P** Western blot analysis to detect the protein expression levels of E-cadherin, N-cadherin, MMP3, Vimentin and Snail in the sh-control and sh-STAMBPL1 group of HCCLM3 cells. **Q** Conducting Western blot analysis to identify the protein expression levels of E-cadherin, N-cadherin, MMP3, Vimentin, and Snail in both the sh-control and sh-STAMBPL1 groups of Hep3B cells

**Fig. 4:**
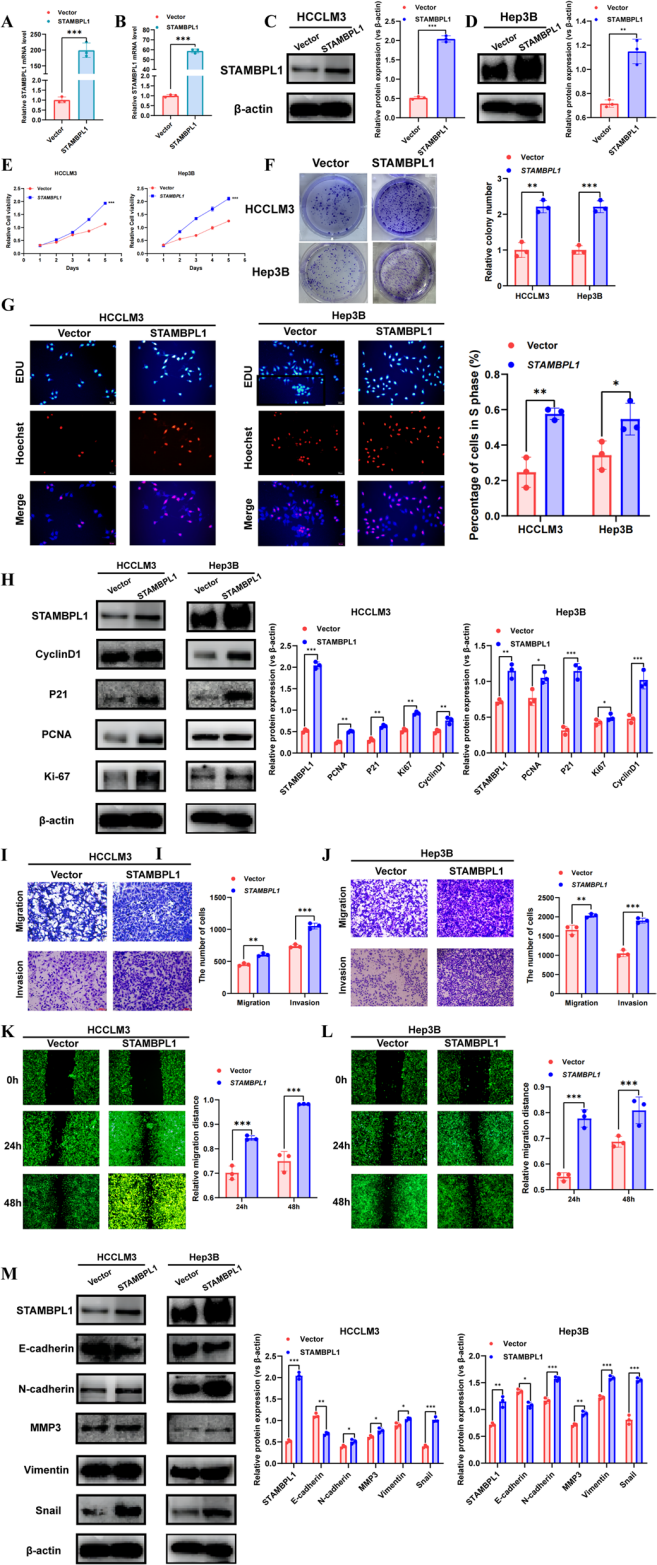
**A** qRT-PCR validation of the overexpression efficiency of STAMBPL1 in HCCLM3 cells. **B** qRT-PCR validation of the overexpression efficiency of STAMBPL1 in Hep3B cells. **C, D** Western blot validation of the overexpression efficiency of STAMBPL1 in HCCLM3 cell and Hep3B cell. **E** The CCK-8 experiment to assess the proliferation efficiency between the vector group and the STAMBPL1 over-expressed group in HCCLM3 and Hep3B cell. **F** The colony formation assay to examine the proliferation efficiency of the vector group and the STAMBPL1 over-expressed group in HCCLM3 and Hep3B cell. **G** The EdU assay to assess the proliferation efficiency of the vector group and the STAMBPL1 over-expressed group in HCCLM3 and Hep3B cell. **H** Western blot analysis to detect the protein expression levels of Cyclin D1, P21, PCNA, and Ki-67 in the vector group and the STAMBPL1 over-expressed group of HCCLM3 and Hep3B cells. **I, J** The transwell assay to assess the migration and invasion efficiency between the vector group and the STAMBPL1 over-expressed group of HCCLM3 and Hep3B cells. **K, L** Wound healing assay to assess the migration efficiency of the vector group and the STAMBPL1 over-expressed group of HCCLM3 and Hep3B cells. **M** Western blot analysis to detect the protein expression levels of E-cadherin, N-cadherin, MMP3, Vimentin and Snail in the vector group and the STAMBPL1 over-expressed group of HCCLM3 and Hep3B cells

The basic expression of STAMBPL1 in HCC cell lines and normal liver cell line LO2 were explored by WB, the results were showed in (Fig. 3A, B). Among various cell lines, HCCLM3 exhibits the highest STAMBPL1 expression, whereas Hep3B demonstrates the lowest STAMBPL1 expression compared to other cell lines. We choose these 2 cell lines for further cell gain–loss function experiments. The efficiency of knockdown STAMBPL1 by shRNA were assessed by qRT-PCR and WB. The collective findings demonstrate that shSTAMBPL1#1 and shSTAMBPL1#2 inhibited the expression of STAMBPL1 effectively (Fig. 3C–F). CCK-8 assays revealed that the inhibition of STAMBPL1 could reduce the proliferation of HCCLM3 and Hep3B (Fig. 3G). In addition, the results from the colony formation experiment demonstrated that the downregulation of STAMBPL1 suppressed the proliferation of hepatocellular carcinoma cells (Fig. 3H). Additionally, the EDU staining also demonstrated that suppression of STAMBPL1 expression could reduce the proportion of proliferative HCC cells (Fig. 3I, J). All cell proliferation experiments illustrated that the expression of STAMBPL1 could influence the proliferation of HCC in vitro. The cells protein was extracted and examined by WB, the results showed that the proliferative-related proteins including CyclinD1, P21, PCNA and Ki-67 were reduced in the shSTAMBPL1 metastable cells (Fig. 3K). The transwell assays were used to evaluate the influence on invasion and metastasis abilities of HCC cells by reducing the expression of STAMBPL1. All results showed a reduce of invasion and metastasis abilities of HCC cells in the group of STAMBPL1 suppression (Fig. 3L, M). The cell scratch assay also confirmed the ability of STAMBPL1 effect on in migration of HCC cells, the results showed the migration abilities of HCCLM3 cells and Hep3B cell all reduced in the shSTAMBPL1#1 and shSTAMBPL1#2 group (Fig. 3N, O). The protein expression of E-cadherin, N-cadherin, MMP3, Vimentin and Snail were reduced in the shSTAMBPL1#1 and shSTAMBPL1#2 group (Fig. 3P). We further constructed stable STAMBPL1 over-expressed cells (including HCCLM3 and Hep3B), and the efficiency of overexpression STAMBPL1 were examined by qRT-PCR and WB, the results showed that overexpression STAMBPL1 can upregulate the STAMBPL1 expression in HCCLM3 cells and Hep3B cells (Fig. 4A–D). The CCK-8 assays showed STAMBPL1 overexpressed group significantly improved the proliferative capacity (Fig. 4E). The colony formation experiments additionally revealed that overexpressing STAMBPL1 heightened the proliferative capacity of hepatocellular carcinoma cells (Fig. 4F). Additionally, the EDU staining also demonstrated that overexpression of STAMBPL1 expression could enhance the proportion of proliferative HCC cells (Fig. 4G). The cells protein was extracted and examined by WB, the results showed that the proliferative related proteins including CyclinD1, P21, PCNA and Ki-67 were upregulated in the STAMBPL1 overexpressed group compared to the vector group in both HCCLM3 cell and Hep3B cell (Fig. 4H). The transwell assays and cell scratch assay both validated the influence of STAMBPL1 on migration and invasion ability of HCCLM3 and Hep3B cells, The migration and invasion ability of HCCLM3 and Hep3B cells was enhanced in the STAMBPL1-overexpressed group (Fig. 4I–J). The cell scratch assay also confirmed the ability of STAMBPL1 effect on in migration of HCC cells, the results showed the migration abilities of HCCLM3 cells and Hep3B cell all enhanced in the STAMBPL1-overexpressed group (Fig. 4K, L). The protein expression of E-cadherin, N-cadherin, MMP3, Vimentin and Snail were elevated in the STAMBPL1-overexpressed group (Fig. 4M).

### STAMBPL1 regulates the WNT/PI3K/ NF-kb signaling pathway in HCC

**Fig. 5:**
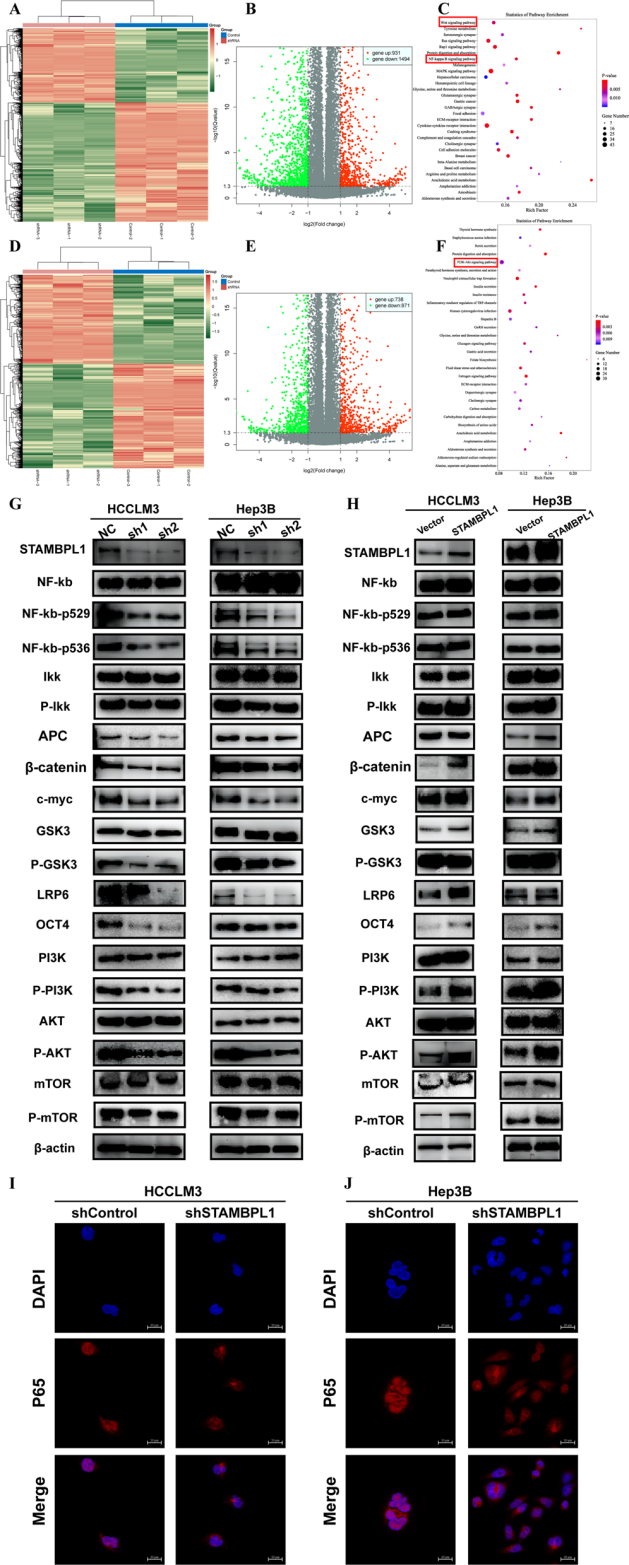
**A** Heatmap illustrating the RNA-seq analysis results of sh-control and sh-STAMBPL1 group in stable transfected HCCLM3 cell line. **B** Volcano plot illustrating the DEGs in the sh-control and sh-STAMBPL1 groups of HCCLM3 cell line. **C** The KEGG enrichment analysis reveals that DEGs are primarily enriched in the WNT and NF-KB pathways in HCCLM3 cell. **D** Heatmap illustrating the RNA-seq analysis results of sh-control and sh-STAMBPL1 groups in stable transfected Hep3B cell line. **E** Volcano plot illustrating the DEGs in the sh-control and sh-STAMBPL1 groups of Hep3B cell. **F** The KEGG enrichment analysis reveals that DEGs are primarily enriched in the PI3K/AKT pathways in Hep3B cell. **G** Validation through Western blot of differential expression of key proteins in the WNT/PI3K/NF-kb signaling pathway between the sh-control and sh-STAMBPL1 group of HCCLM3 cell and Hep3B cell. **H** Validation through Western blot of differential expression of key proteins in the WNT/PI3K/NF-kb signaling pathway between the vector group and the STAMBPL1 over-expressed group of HCCLM3 and Hep3B cells. **I** Detection using cell immunofluorescence (ICC/IF) revealed that knocking down STAMBPL1 inhibited the nuclear translocation of the P65 protein in HCCLM3 cell. **J** Detection using cell immunofluorescence (ICC/IF) revealed that knocking down STAMBPL1 inhibited the nuclear translocation of the P65 protein in Hep3B cell

In order to explore potential signaling pathway and biological functions correlated with the expression of STAMBPL1 in HCC, HCCLM3 cell lines transfected with shRNA were examined by the next generation sequencing (NGS). In the results of NGS, there were 931 up-regulated DEGs and 1494 down-regulated DEGs (Fig. 5A, B). The KEGG analysis of these DEGs showed several signaling pathway correlated with STAMBPL1, especially the WNT signaling pathway and NF-kb signaling pathway (Fig. 5C). Besides, in order to further exploit potential mechanism of STAMBPL1, we transfected Hep3B cell lines and examined by the next generation sequencing (NGS). In the results of NGS, there were 738 up-regulated DEGs and 871 down-regulated DEGs (Fig. 5D, E). The KEGG analysis of these DEGs showed several signaling pathway correlated with STAMBPL1 and PI3K/AKT signaling pathway emerged as one of the foremost and impactful pathways (Fig. 5F). We used the WB experiments to investigate the correlation between STAMBPL1 and WNT/PI3K/ NF-kb signaling pathway in HCC cell lines. In the result of WB, the inhibition of STAMBPL1 via shRNA could suppress the activation of WNT/PI3K/ NF-kb signaling pathway (Fig. 5G). The over-expression of STAMBPL1 via shRNA could also enhance the key protein expression of WNT/PI3K/ NF-kb signaling pathway (Fig. 5H). These experiments hinted that the expression of STAMBPL1 could regulate the activation of WNT/PI3K/ NF-kb signaling pathway in HCC. The ICC/IF experiment showed the expression of STAMBPL1 could mediate the P65 protein to transfer to the nucleus (Fig. 5I, J). It further verified the expression of STAMBPL1 could regulate the activation of NF-kb signaling pathway in HCC.

### STAMBPL1 directly interacts with TRAF2 and stabilize its protein stability

**Fig. 6:**
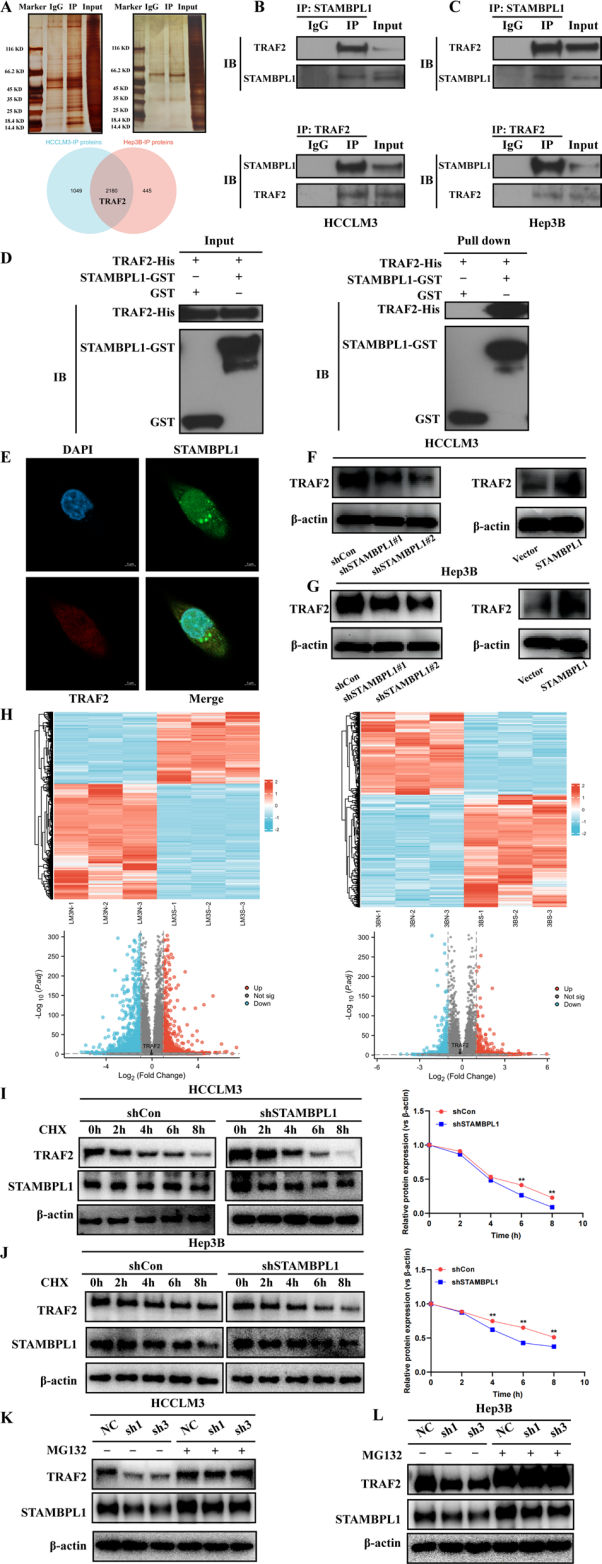
**A** Through mass spectrometry (MS) analysis, proteins interacting with STAMBPL1 in HCCLM3 and Hep3B cells were identified, among which TRAF2 is one of the proteins obtained in the intersection. **B** Immunoprecipitation analysis identified an interaction between STAMBPL1 and TRAF2 in HCCLM3 cells. **C** Immunoprecipitation analysis identified an interaction between STAMBPL1 and TRAF2 in Hep3B cells. **D** Through GST-pull down analysis, a direct interaction between STAMBPL1 and TRAF2 was identified in HCCLM3 cells. **E** Cell Immunofluorescence analysis detected co-localization of STAMBPL1 and TRAF2 in the cytoplasm. **F** WB analysis revealed that in HCCLM3 cells, the expression level of TRAF2 decreases in the STAMBPL1 knockdown group and increases in the STAMBPL1 overexpression group. **G** WB analysis revealed that in Hep3B cells, the expression level of TRAF2 decreases in the STAMBPL1 knockdown group and increases in the STAMBPL1 overexpression group. **H** Heatmap and volcano plot illustrate that TRAF2 shows no significant differential expression in the obtained RNA-seq results. **I** After treating HCCLM3 cells with CHX (0.2 mg/ml), the TRAF2 protein levels were assessed over specified time points following the downregulation of STAMBPL1. **J** After treating Hep3B cells with CHX (0.2 mg/ml), the TRAF2 protein levels were assessed over specified time points following the downregulation of STAMBPL1. **K** After treatment with 20 μM MG132, the levels of TRAF2 were assessed in HCCLM3 cells following the downregulation of STAMBPL1. **L** After treatment with 20 μM MG132, the levels of TRAF2 were assessed in Hep3B cells following the downregulation of STAMBPL1

The results of mass spectrometry analysis to identify the proteins conjugated with STAMBPL1 in HCCLM3 cell line and Hep3B cell line were showed in Fig. 6A. About 3229 proteins were enriched in IP group of HCCLM3 cell result and 2625 proteins were enriched in IP group of Hep3B cell result, TRAF2 is one protein in the intersection of HCCLM3-IP group and Hep3B-IP group, and previous studies suggested TRAF2 is correlated with WNT/PI3K/NF-kb signaling pathway in various pathological process. The CO-IP experiment was further utilized to identify the interaction of STAMBPL1 and TRAF2 in HCCLM3 and Hep3B cell line, and the result showed that STAMBPL1 can interact with TRAF2 in both HCCLM3 and Hep3B cell line (Fig. 6B, C). At the same time, the GST-pull down assay was used to verify STAMBPL1 directly interacts with TRAF2 (Fig. 6D).

The Immunofluorescence co-localization assay indicated that STAMBPL1 and TRAF2 are co-localized in the cytoplasm (Fig. 6E). The protein expression of TRAF2 showed a substantial reduction in both the shSTAMBPL1#1 and shSTAMBPL1#2 groups (Fig. 6F), and the TRAF2 expression was increased in the STAMBPL1 over-expressed group (Fig. 6G). Combing the previous RNA-seq result, it was found that TRAF2 had no significant difference between the control group and shSTAMBPL1 group (Fig. 6H). To investigate the impact of STAMBL1 on the protein half-life of TRAF2, the cycloheximide chase (CHX) assay was conducted and the result showed that the protein degradation rate is faster in shSTAMBPL1 group compared with shControl group by using HCCLM3 cells (Fig. 6I) and Hep3B cells (Fig. 6J). When the MG132 is used to inhibit the protease degradation system, the protein expression of TRAF2 did not change with the expression of STAMBPL1 in either HCCLM3 cell and Hep3B cell (Fig. 6K, L).

### STAMBPL1 stabilizes TRAF2 through deubiquitinating the K63 ubiquitin site

**Fig. 7:**
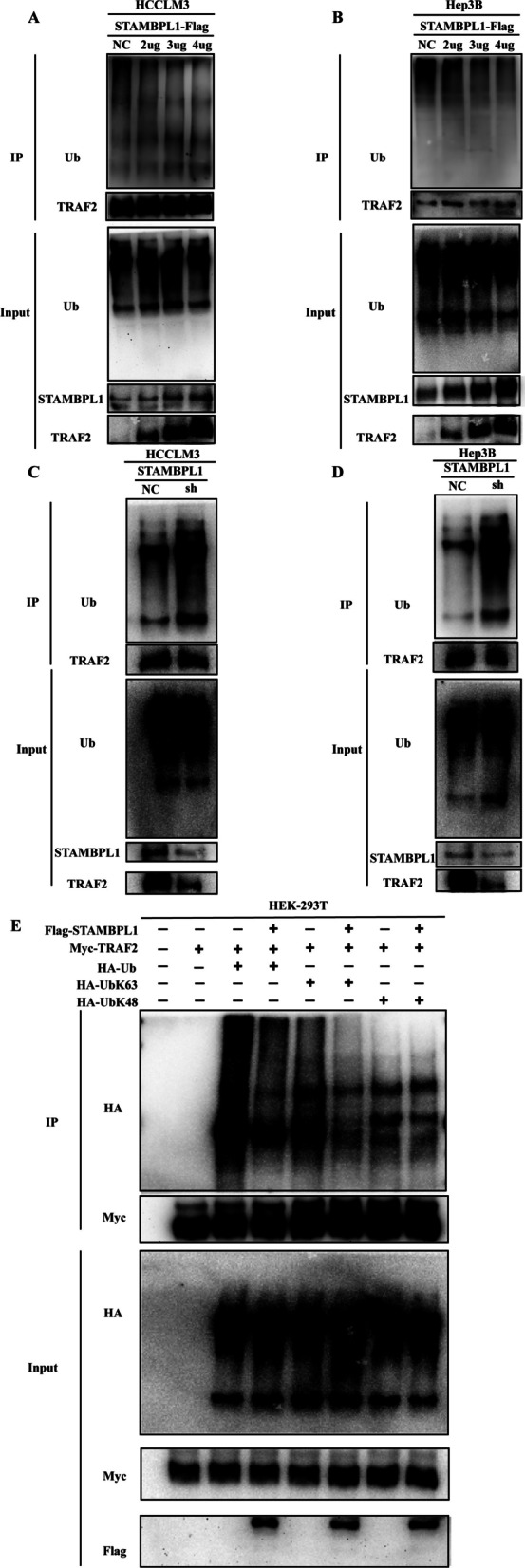
**A** In HCCLM3 cells, gradient overexpression of STAMBPL1 resulted in a reduced ubiquitination level of TRAF2. **B** In Hep3B cells, gradient overexpression of STAMBPL1 resulted in a reduced ubiquitination level of TRAF2. **C** In HCCLM3 cells, the ubiquitination level of TRAF2 elevated following the downregulation of STAMBPL1. **D** In Hep3B cells, the ubiquitination level of TRAF2 elevated following the downregulation of STAMBPL1. **E** IP and WB experiments revealed that STAMBPL1 mediates deubiquitination modification of TRAF2 through K63 ubiquitin sites

The Flag-STAMBPL1 plasmid were well constructed and transferred to HCCLM3 and Hep3B cell, including (Control, Flag-STAMBPL1-2ug, Flag-STAMBPL1-3ug, Flag-STAMBPL1-4ug), The findings indicated that elevated STAMBPL1 levels led to a decrease in the ubiquitination levels of TRAF2 in both HCCLM3 and Hep3B cells (Fig. 7A, B). Meanwhile, the ubiquitination level of TRAF2 was also detected in control group and shSTAMBPL1 group, and it showed that the inhibition of STAMBPL1 can increasing the ubiquitination level of TRAF2 in both HCCLM3 and Hep3B cell (Fig. 7C, D). The plasmid truncates of ubiquitin was constructed, which includes HA-K48, HA-K63 plasmid. The IP assay indicated that the deubiquitinating enzyme activity of STAMBPL1 on TRAF2 need the combination of the K63 site of ubiquitin chain (Fig. 7E).

### The specific binding sites of interaction between STAMBPL1 and TRAF2

**Fig. 8:**
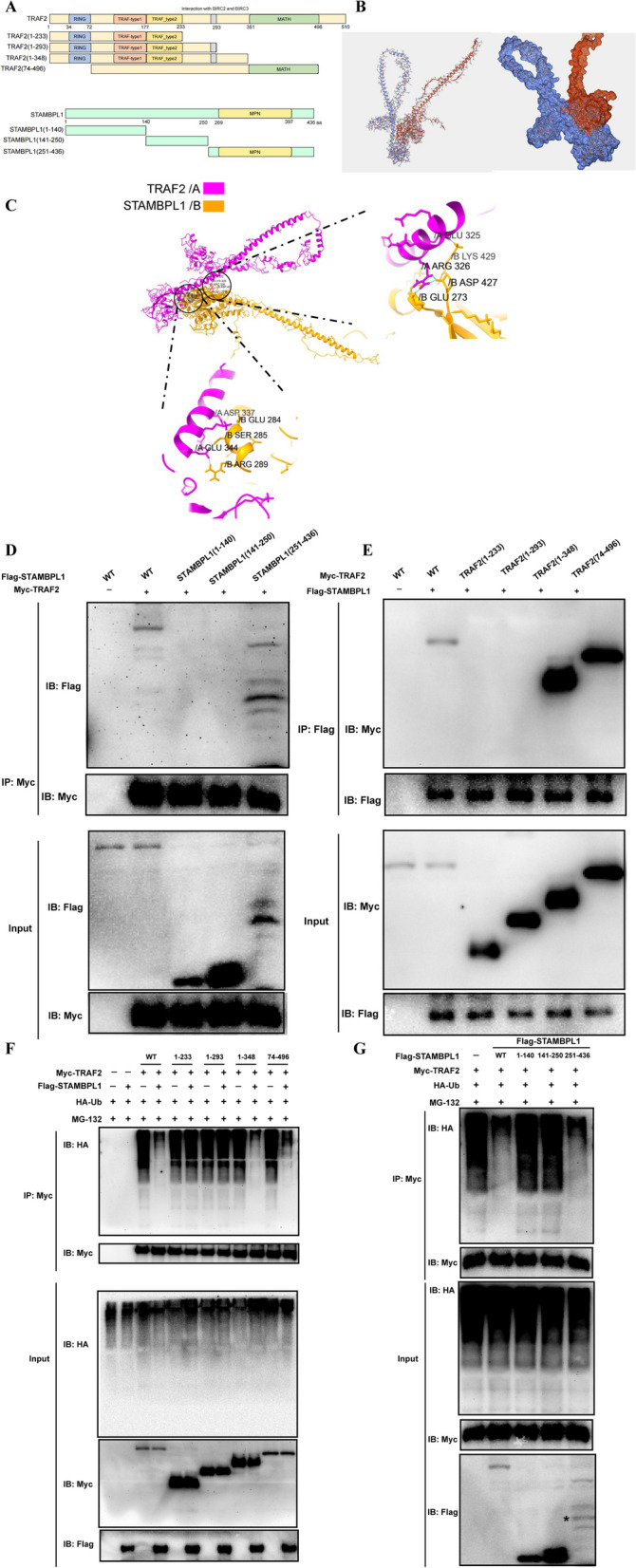
**A** Schematic diagram illustrating the constructed truncated plasmids TRAF2(1-233), TRAF2(1-293), TRAF2(1-348), TRAF2(74-496), STAMBPL1(1-140), STAMBPL1(141-250), and STAMBPL1(251-436). **B** Molecular docking reveals the three-dimensional protein structures of STAMBPL1 and TRAF2, as well as their binding interactions with each other. **C** Molecular docking reveals the specific binding sites of interaction between STAMBPL1 and TRAF2. **D** The IP results demonstrate that specific sites on STAMBPL1 interacts with TRAF2. **E** The IP results reveal the specific sites on TRAF2 interacts with STAMBPL1. **F** The IP results demonstrate that STAMBPL1 can only mediate deubiquitination modification on TRAF2(1-348) and TRAF2(74-496). **G** The IP results demonstrate that STAMBPL1(251-436) is capable of mediating deubiquitination modification on TRAF2

The proteins TRAF2 and STAMBPL1 are truncated as shown in Fig. 8A, and the corresponding plasmids (TRAF2(1-233), TRAF2(1-293), TRAF2(1-348), TRAF2(74-496), STAMBPL1(1-140), STAMBPL1(141-250), and STAMBPL1(251-436)) are constructed for transfection into 293 T cells.

Utilizing molecular docking simulations to predict the structures of proteins TRAF2 and STAMBPL1, as shown in Fig. 8B. Employing molecular docking simulations further to anticipate the precise binding locations of TRAF2 and STAMBPL1 proteins, as depicted in Fig. 8C. STAMBPL1 and TRAF2 may interact through binding at STAMBPL1 (ARG 326, GLU325, GLU344, ASP337 sites) and TRAF2 (GLU 273, ASP 427, LYS 429, ARG 289, SER 285, GLU 284). IP assays confirmed the interaction between the STAMBPL1 (251-436) fragment and TRAF2 (Fig. 8D). The interaction between the TRAF2 (1-348), TRAF2 (74-496) fragments, and STAMBPL1 was verified (Fig. 8E). Simultaneously, through IP experiments, it was further discovered that STAMBPL1 can interact with the TRAF2 (1-348), TRAF2 (74-496) fragments and undergo deubiquitination modification (Fig. 8F). The STAMBPL1 (251-436) fragment can undergo deubiquitination modification of TRAF2 (Fig. 8G).

### TRAF2 reversed the tumorigenic activity of STAMBPL1 in HCC cells

**Fig. 9:**
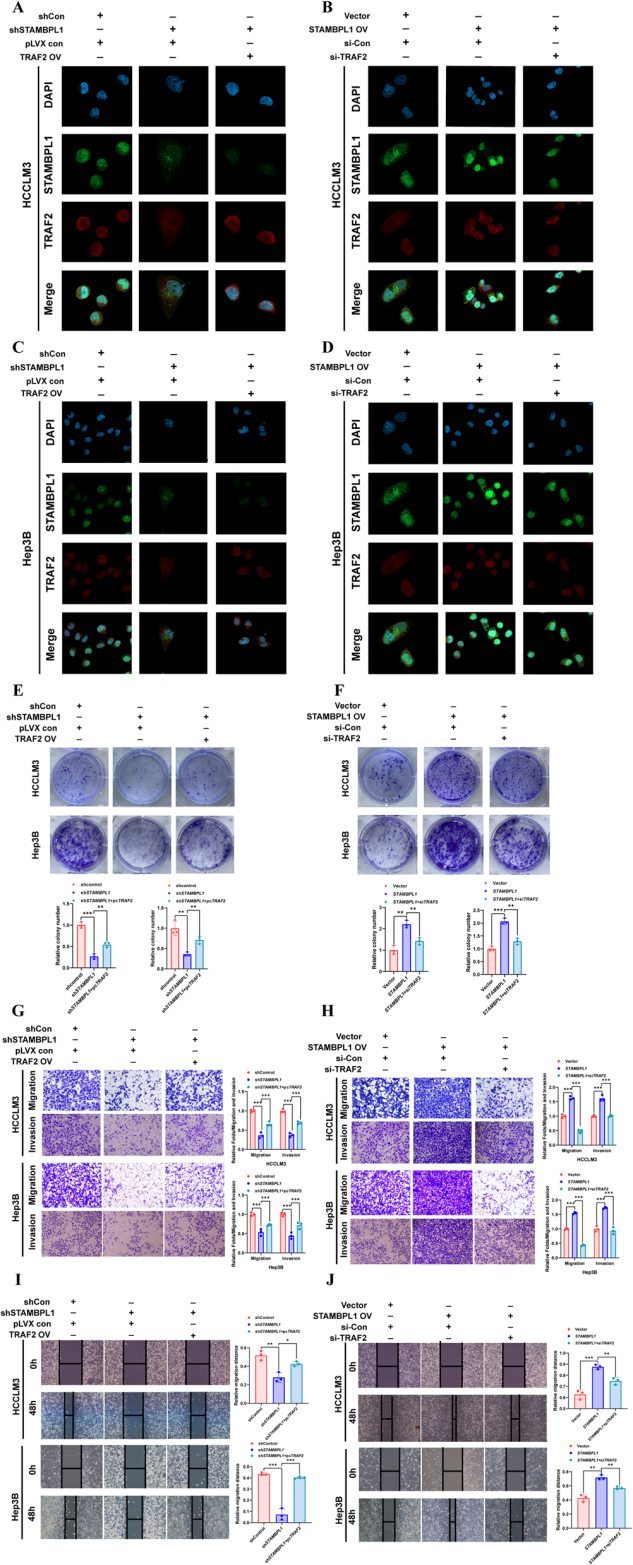
**A, B** Cell immunofluorescence assay was used to analyze STAMBPL1 and TRAF2 expression in HCCLM3 cell subjected to either TRAF2 knockdown or overexpression within STAMBPL1-interfering HCCLM3 cell. **C, D** Cell immunofluorescence assay was used to analyze STAMBPL1 and TRAF2 expression in Hep3B cell subjected to either TRAF2 knockdown or overexpression within STAMBPL1-interfering Hep3B cell. **E, F** The colony formation assays were conducted on HCCLM3 cell and Hep3B cell to investigate changes in cell proliferation following alterations in TRAF2 and STAMBPL1 expression. **G, H** Transwell assays were conducted on HCC cells to assess changes in cell migration and invasion following alterations in TRAF2 and STAMBPL1 expression. **I, J** Wound-healing assays were conducted on HCC cells to assess changes in cell migration following alterations in TRAF2 and STAMBPL1 expression

**Fig. 10:**
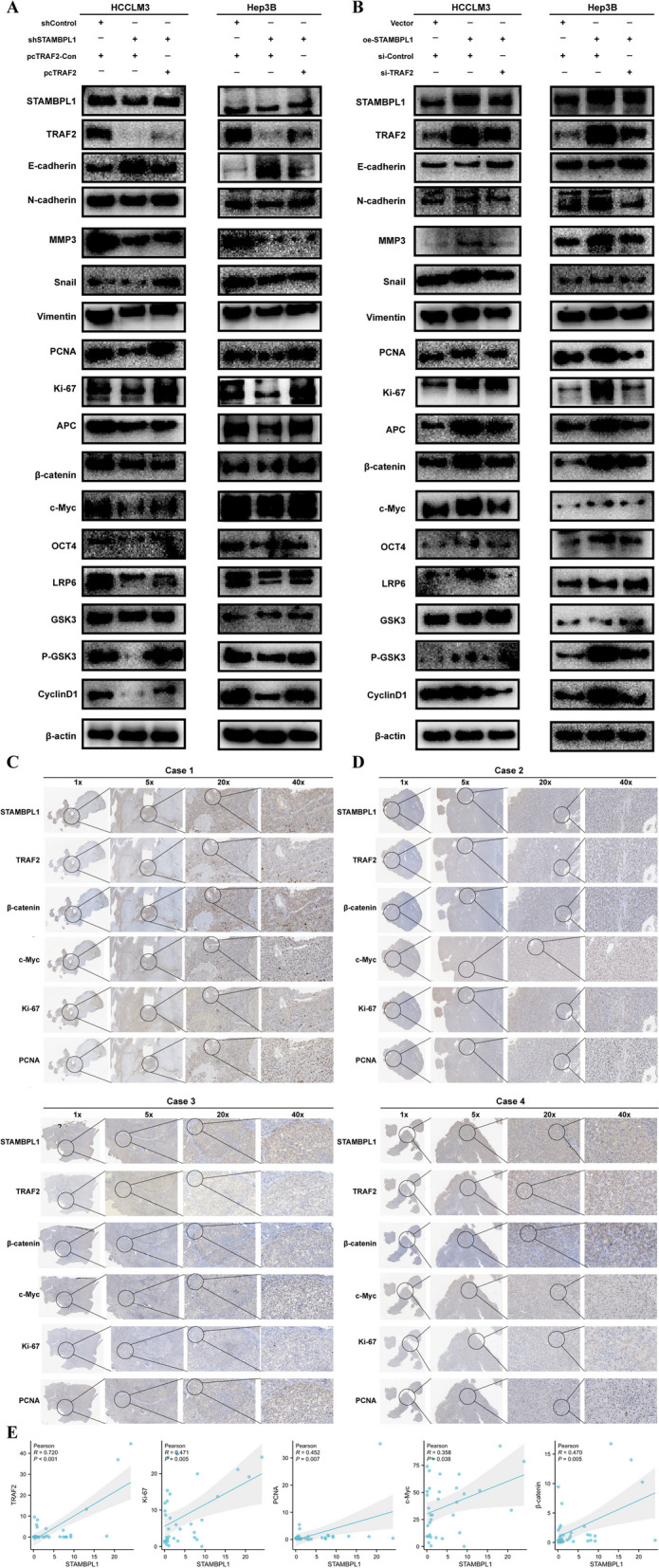
**A, B** HCC cells, subjected to either TRAF2 knockdown or overexpression, were analyzed for EMT and WNT pathway related protein expression in STAMBPL1-interfering cells using western blotting. **C, D** The Immunohistochemistry results show a significant positive correlation between the expression of STAMBPL1 and TRAF2, as well as key proteins in the WNT pathway, in human HCC samples. **E** The scatter plot illustrates a significant positive correlation in the expression of STAMBPL1 with TRAF2 and key proteins in the WNT pathway

The cell immunofluorescence staining presented the differential expression of STAMBPL1 and TRAF2 in the various groups (Fig. 9A–D). In order to clarify the effect of TRAF2 in the tumorigenic activity of STAMBPL1 in HCC, the effect of TRAF2 knockdown or overexpression on the carcinogenesis of STAMBPL1 stable knockdown or overexpression HCCLM3 cell line and Hep3B cell line were detected. The results from the colony formation assay suggested that TRAF2 can reverse the pro-proliferative ability of STAMBPL1 in HCC cells (Fig. 9E–F). The transwell assay showed the pro-migration and pro-invasion ability of STAMBPL1 can also be reversed in HCC cells (Fig. 9G, H). The cell wound scratch assay further confirmed that the pro-migration ability of STAMBPL1 can also be reversed in HCC cells (Fig. 9I, J). The cell protein was extracted and detected by WB, the result showed the upregulation or downregulation of key protein of WNT/PI3K/ NF-kb signaling pathway can be reverse through TRAF2 knockdown or overexpression (Fig. 10A, B). The IHC staining result indicate that the STAMBPL1 expression had a positive correlation with TRAF2 expression in human HCC specimens (cor = 0.72, *P* < 0.001), and the STAMBPL1 expression has positive association with proliferative and metastatic related protein (Ki-67, cor = 0.471, *P* = 0.005; PCNA, cor = 0.452, *P* = 0.007). The key pathway related protein of WNT signaling pathway had positive correlation with STAMBPL1 expression (c-Myc, cor = 0.358, *P* = 0.038; β-catenin, cor = 0.470, P = 0.005). The representative immunohistochemical images and scatter diagram were presented in Fig. 10C–E.

### STAMBPL1 positively mediated tumor growth and TRAF2 reversed its tumorigenic activity in vivo

**Fig. 11:**
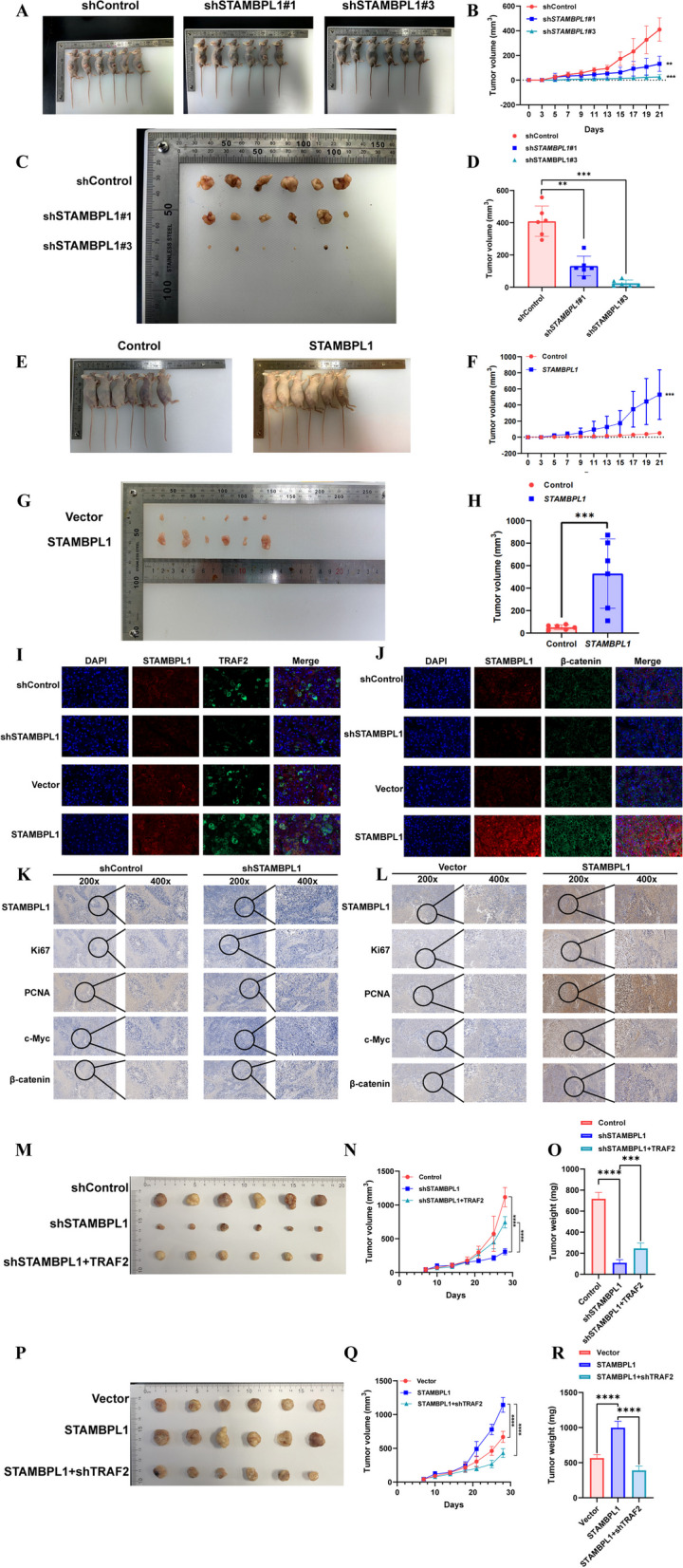
**A** Representative images depict nude mice following subcutaneous injection of HCCLM3 cells (n = 6). **B** The tumor volume curves were plotted based on the measured tumor diameters. **C** Representative images depict the excised tumors following subcutaneous injection of HCCLM3 cells into nude mice. **D** Representative images depict the volume of the tumor at the time of excision. **E** Representative images depict nude mice following subcutaneous injection of Hep3B cells (n = 6). **F** The tumor volume curves were plotted based on the measured tumor diameters. **G** Representative images depict the excised tumors following subcutaneous injection of Hep3B cells into nude mice. **H** Representative images depict the volume of the tumor at the time of excision. **I** Immunofluorescence images of the subcutaneous tumor tissue show that STAMBPL1 and TRAF2 co-localize, and there is a significant positive correlation between the expression levels of TRAF2 and STAMBPL1. **J** Immunofluorescence images of the subcutaneous tumor tissue show that there is a significant positive correlation between the expression levels of β-catenin and STAMBPL1. **K, L** Immunohistochemistry experiments reveal a significant positive correlation between STAMBPL1 expression and the expression of Ki-67, PCNA, c-Myc, and β-catenin in subcutaneous tumor-bearing mice. **M–O** A xenograft tumor formation assay was conducted using HCCLM3 cells that were transfected with shControl, shSTAMBPL1, and subsequently reconstituted with TRAF2. **M** The tumors were visually represented in the provided gross images. **N** The growth curve of subcutaneous tumors in nude mice. **O** The weight of subcutaneous tumors in mice. **P–R** Hep3B cells were subjected to a xenograft tumor formation assay after transfection with either a vector or STAMBPL1, followed by reconstitution with sh-TRAF2. **P** The tumors were visually represented in the provided gross images. **Q** The growth curve of subcutaneous tumors in nude mice. **R** The weight of subcutaneous tumors in mice

In order to further verify the results, the orthotopic tumor model in nude mice was established. The results indicated a substantial reduction in tumor growth rate and volume in nude mice upon downregulation of STAMBPL1 (Fig. 11A–D). Besides, upregulation of STAMBPL1 could significantly enhance the tumor growth rate and volume in nude mice (Fig. 11E–H). The multiple immunofluorescences of orthotopic tumor showed a higher expression of STAMBPL1, TRAF2 and β-catenin in the negative control group compared with the shSTAMBPL1 group were detected, and the higher expression of STAMBPL1, TRAF2 and β-catenin in the STAMBPL1 overexpressing group was also detected, as opposed to the negative control group (Fig. 11I, J). In the STAMBPL1 overexpressing group, the IHC results indicated higher expression levels of Ki-67, PCNA, c-Myc, and β-catenin, while the shSTAMBPL1 group exhibited lower expression levels for these markers (Fig. 11K, L). All these results hinted that the STAMBPL1 positively mediated tumor growth in vitro. TRAF2 can reverse the tumorigenic activity of STAMBPL1 in vitro at the same time (Fig. 11M–R).

### Molecular docking analysis of the interaction between STAMBPL1 and first-line drugs for HCC

The study employed molecular docking techniques to analyze the interaction between the STAMBPL1 protein and commonly used first-line drugs for clinical liver cancer (including sorafenib, regorafenib, lenvatinib, and cabozantinib). The results revealed that the STAMBPL1 protein can stably bind to sorafenib, regorafenib, lenvatinib, and cabozantinib, with binding energies of −12.4472 kcal/mol, −21.769 kcal/mol, −24.1708 kcal/mol, and −36.5506 kcal/mol (Supplemental Figure S2A–D), respectively. The overall message of the study was represented by the graphical abstract (Supplemental Figure S3).

## Discussion

HCC has caused millions of death toll around the world because of the high morbidity and recurrence rate [16]. Although great progresses about the diagnoses and therapy of HCC have been created in the past 20 years, the prognosis of patients still be unsatisfactory [17]. In several study, plenty of deubiquitinase ligases were demonstrated to play essential roles in the carcinogenesis or progression of HCC. For example, Loss of the deubiquitinase OTULIN has been shown to promote HCC in an mTOR-dependent manner. This suggests that OTULIN normally acts to suppress HCC, and its loss can lead to cancer progression [18]. EIF3H has been found to promote HCC progression by stabilizing OGT (O-GlcNAc transferase) and inhibiting ferroptosis, a form of regulated cell death. By preventing ferroptosis, EIF3H may contribute to the survival of cancer cells [19]. USP39, which was in conjunction with the E3 ligase TRIM26, regulates the ubiquitination level of ZEB1 and determines the progression of HCC [20]. In addition, USP27 has been shown to stabilize SETD3, which enhances cell proliferation and contributes to HCC progression [21]. These findings highlight the diverse roles that deubiquitinases play in HCC, either by promoting or suppressing the disease, and suggest that they could be potential targets for therapeutic intervention in HCC.

In the study, we identified one oncogene gene (STAMBPL1) which promote the proliferation and metastasis of HCC. Especially STAMBPL1 could modulate the stability of TRAF2 protein and activate WNT/PI3K/NF-kb signalling pathway. STAMBPL1 (STAM Binding Protein Like 1) is a genetic element responsible for encoding a protein engaged in cell signaling and endocytosis processes. Recent studies indicate that STAMBPL1 plays a role in the advancement of various cancer types, such as gastric, breast, prostate, and colorectal cancers. STAMBPL1 expression was found to be significantly upregulated in gastric cancer compared to adjacent tissue, and the expression levels increased with advanced stages [22]. Especially in gastric cancer cells, long non-coding RNA NEAT1 could regulate STAMBPL1 expression and promote its malignant behavior [14]. STAMBPL1 depletion was found to induce apoptosis by promoting the degradation of the protein XIAP, suggesting that targeting STAMBPL1 might offer a promising therapeutic strategy for prostate cancer [23]. STAMBPL1 knockout or correction of the heterozygous STAMBPL1 mutation in a human colon cancer cell could suppress xenograft tumor growth [24]. These studies hinted a potential prognostic predictive value and therapeutic capacity of STAMBPL1 in malignancies, our study identify its oncogene effect on HCC, STAMBPL1 promote proliferation, invasion, and migration of HCC cells through stabilize TRAF2 via deubiquitinating K63 Ub chain activating WNT/PI3K/NF-kb signalling pathway. Thus, these studies may provide a new target to inhibit the proliferation or metastasis of HCC.

TRAF2 is involved in the signaling pathways of tumor necrosis factor receptors (TNFRs), which can induce either cell survival or apoptotic cell death. Studies have shown that TRAF2 can interact with other proteins, such as TRAF6, which can affect the downstream immune signaling in the various cellular processes [25]. The dynamics of TRAF2 are influenced by the length of its tail, with longer tails affecting the dynamics of the globular regions in the protein's C-terminal head [26]. Molecular dynamics studies showed that the monomer–trimer equilibrium of TRAF2 is crucial for several functions, including receptor recognition, membrane binding, and hetero-oligomerization [26, 27]. In cancer research, TRAF2 has been implicated in the biological behavior of various cancers, including bladder cancer [28] and lung cancer [29]. TRAF2, particularly, has demonstrated elevated expression at both protein and mRNA levels in hepatocellular carcinoma (HCC) tissues. The heightened TRAF2 expression correlates with an unfavorable prognosis for HCC patients, suggesting a potential involvement of increased TRAF2 expression in HCC tumorigenesis [30]. The latest research further showed that TRAF2 deficiency increases ROMO1 expression and stimulate the NAD + /SIRT3/SOD2 signaling pathway to promote the ROS production of HCC cells and its mitochondrial dysfunction [31]. Our study further highlights the carcinogenic role of TRAF2 in HCC and found that STAMBPL1, as a deubiquitinase that stabilizes its protein structure, plays an important oncogene in HCC.

The WNT, PI3K/AKT, and NF-κB pathways are all critical signaling pathways involved in various cellular processes, including cell proliferation, survival, and differentiation. The dysregulation is often associated with the onset and advancement of cancer. In CRC, the WNT pathway interacts with the PI3K/AKT pathway during the epithelial-to-mesenchymal transition (EMT) process, a critical step in cancer progression and metastasis [32]. The PI3K/AKT pathway is also overactivated in bladder cancer, promoting cell proliferation, survival, and metastasis, and the NF-kB pathway inhibitor (NFKBIZ) can inhibit tumor cell proliferation through the PTEN/PI3K/Akt signaling pathway [33]. NLRP12, an inhibitory modulator of the NF-kB pathway, has been identified as a regulator of the WNT/β-catenin pathway in colorectal cells, suggesting a novel clinical therapeutic target for CRC patients [34].

To find target to destroy the proliferation or metastasis might be effective way to treat HCC. finding deubiquitinating enzymes as new target to inhibit the proliferation or metastasis could be a novel research direction. In our study, the expression of STAMBPL1 in HCC samples was higher than in normal samples, which might reminder a possible regulation between STAMBPL1 and HCC. High expression of STAMBPL1 showed worse OS rates according to the K-M curves, which revealed a prognostic value of HCC. Subsequent observations revealed that the reduction of STAMBPL1 in hepatocellular carcinoma cell lines led to the inhibition of cell proliferation, invasion, and metastasis. The results of nude mice also demonstrated the oncogenic function of STAMBPL1. Additionally, STAMBPL1 was also demonstrated to regulate the stability of TRAF2 via activating WNT/PI3K/NF-kb signalling pathway in HCC. These findings indicate that STAMBPL1 could serve as a promising prognostic biomarker and therapeutic target.

In summary, the heightened expression of STAMBPL1 not only impacts the proliferation and metastasis of HCC cells but also serves as an ominous predictor of poor prognosis in HCC patients, suggesting that exploring STAMBPL1-based research could offer promising avenues for enhancing the prognosis of HCC patients.

## Methods

### Gene expression analysis and prognostic analysis

Genetic mRNA data of 374 clinical samples and corresponding clinical information were gained from The Cancer Genome Atlas (TCGA) database (https://portal.gdc.cancer.gov/). In addition, the mRNA data and clinical prognostic data of HCC samples and paracancerous samples were downloaded from GEO database (https://www.ncbi.nlm.nih.gov/geo/), and GSE46408, GSE112790, GSE121248 datasets were included. Gene expression analysis of STAMBPL1 in databases were performed by “limma” package via R software 4.2.2. The prognostic value of STAMBPL1 was analyzed by Kaplan–Meier Plotter website (https://kmplot.com/analysis/).

### Clinical samples and RNA microarray

HCC samples and corresponding paracancerous samples were collected from the department of general surgery, Changzhou Second people’s hospital. Each sample were obtained after patients provided their written informed consent. Samples were frozen in liquid nitrogen immediately or stored in −80 ℃ after surgical resection for further RNA extraction, or fixed in 4% paraformaldehyde and embedded in paraffin for tissue microarray (TMA) construction. We sent the total RNA of HCC samples and corresponding paracancerous samples from 3 patients to Riobio company and identified the DEGs (|log2FC > 1| and *p* < 0.05) between HCC samples and paracancerous samples via RNA microarray.

### RNA extraction and quantitative real time PCR (qRT-PCR)

According to the manufacturer’s instructions, Total RNA was isolated using Total RNA TriPure Isolation Reagent Kit (BioTeke, Beijing, China). Total RNA was reverse transcribed to cDNA by utilizing Vazyme HiScript II Q RTSuper mix (Vazyme, China). qRT-PCR were performed by the real-time PCR system (ABI, Waltham,MA, USA). The expression of each gene was measured and calculated through the 2^−ΔΔCT^ method. All primer sequences were listed in Supplementary Table S1.

### Cell lines, reagents, and transfection

In this study, the human HCC cell lines Huh7, HepG2, Hep3B, HCCLM3 and normal liver cell line LO2 were gained from the Chinese Academy of Science Cell Bank (Shanghai, China). All cell lines were cultured in DMEM (Gibco, USA) supplemented with 1% streptomycin/penicillin and 10% fetal bovine serum. All cells in this study were cultured at 37 °C and a CO_2_ concentration of 5%. All vectors were acquired from Shanghai Genechem Co. Ltd (Shanghai, China). The Hep3B and HCCLM3 cell line were transfected with STAMBPL1 short hairpin RNA (shRNA) lentiviral vectors and control vectors. The primer sequence of STAMBPL1 is shown in Supplementary Table S1. All shRNA sequences were listed in Supplementary Table S2. The transfected cells were selected by adding puromycin to the culture medium for 5 days.

### Immunohistochemistry (IHC), immunofluorescence (IF), and western blotting (WB)

The detailed processes of IHC and IF were described in supplementary materials. In addition, the manufacturer’s protocol of WB was performed as described [35]. All antibody used in the study were listed in Supplementary Table S3.

### Cell proliferation assay

In order to evaluate the proliferation, 2 × 10^3^ cells that transfected with shRNA of STAMBPL1 and control vectors were incubated in 96-well plates, and at the prescribed time, the OD value below 450 nm was measured by spectrophotometer (BioTer EPOCH, USA) after incubated in a cell counting kit-8 (CCK-8) assay (Beyotime Biotechnology, Shanghai, China) every day. Colony formation was also used to assess the capacity of proliferation. After transfected with shRNA, cells were incubated in 6-well plates for a period of 14 days. Then the cells were fixed by methanol for 15 min and stained by crystal violet for 30 min. A 5-ethynyl-2′-deoxyuridine (EdU) assay was also utilized to assess the proliferation of HCC cells. 1 × 10^5^ cells/well were cultured in 24-well plates for 24 days. 15 µM EdU reagent (Beyotime, Shanghai, China) was added to each well and incubated in 37 °C for 2 h. All cells were visualized by fluorescence microscope (Nikon, Japan). Finally, we calculated the ratio of cells with EDU positive.

### Cell migration and invasion assay

The cell migration and invasion were evaluated via Transwell assays with or without matrigel. 5 × 10^4^ cells were seeded into the upper chamber without serum, and 700ul complete medium was presented in the lower chamber. The fixation and staining process followed a procedure similar to that employed in colony formation assays. The cell wound scratch assay was performed as reported previously [35].

### Biological function enrichment analyses

The different expressed genes (DEGs) between different samples were identified through “limma” package (|log2FC > 1| and *p* < 0.05). Potential biological functions and pathways correlated with DEGs were explored by Gene Ontology (GO) analysis, Kyoto Encyclopedia of Genes and Genomes (KEGG) analysis, Gene Set Enrichment Analysis (GSEA), and Gene Set Variation Analysis (GSVA). GO and KEGG analyses were performed by DAVID website database (http://david.abcc.ncifcrf.gov/). The results of GSEA and GSVA were acquired by using “limma”, “GSEABase”, “GSVA” packages.

### Coimmunoprecipitation (CO-IP) and mass spectrometry (MS)

The cells were washed by PBS and then added the lysis buffer with 1 mM PMSF. The blend was allowed to incubate at 4 °C for 20 min, and the resultant lysate was then gathered into 1.5 mL tubes. Subsequently, the mixture underwent ultrasonication for 5 s, followed by centrifugation at 14,000 g for 15 min. The supernatant was collected and supplemented with 5 μg of the primary antibody (STAMBPL1 or IgG), followed by a 12-h incubation period. Subsequently, protein A and protein agarose beads were incubated with the lysate for 3 h, followed by three wash cycles. Subsequently, the lysate was boiled in 1 × SDS loading buffer for 5 min. The proteins obtained from the co-immunoprecipitation results were utilized in Western blot assays. The products of Co-IP were sent to GeneCreate Biological Engineering (Wuhan, China) for further mass spectrometry analysis.

### GST-pull down assay

The GST pull-down assay is a fast and intuitive in vitro method used to analyze protein–protein or protein–ligand interactions. The assay involves a "bait" protein, which is a GST-fused protein expressed in an E. coli host or a baculovirus expression system, and a "prey" protein, which is a putative binding partner protein or other ligand molecule.

### Tumor growth assay in vivo

The Four-Five weeks old nude mice were obtained from the Changzhou Kavins Laboratory Animal (Cavens Biogle, China) and were fed in the specific pathogen-free animal laboratory. 4 × 10^6^ HCC cells transfected with shRNA were injected into the axilla of nude mice. The sizes of tumor were measured every two days until the 21nd days. The formula to calculate tumor volume is: 0.5 × (smaller diameter)^2^ × (larger diameter).

## Molecular docking analysis

Molecular docking studies were conducted to investigate the interaction between the STAMBPL1 protein and clinically relevant first-line drugs for HCC. The protein structure of STAMBPL1 was obtained from RCSB ProteinData Bank (PDB, http://www.pdb.org), and the three-dimensional structures of sorafenib, regorafenib, lenvatinib, and cabozantinib were retrieved from the PubChem database. The molecular docking simulations were performed using discovery studio. The binding sites on the STAMBPL1 protein were predicted and validated prior to the docking simulations. The results were analyzed in terms of binding affinity, and the stability of the protein-drug complexes was assessed based on the calculated binding energies. Furthermore, graphical representations of the molecular interactions, such as 2D ligand interaction diagrams, were generated to visualize the key binding features. The entire process was repeated with rigorous parameters to ensure the reliability of the docking results. The binding energies for each interaction were recorded, and statistical analyses were performed to draw meaningful conclusions regarding the potential binding of STAMBPL1 with sorafenib, regorafenib, lenvatinib, and cabozantinib.

### Statistical analysis

All statistical analyses were performed by Graphpad Prism 8 and R software 4.0.0. All numerical results were obtained through three repeated experimental trials. The t-test was utilized to analyze the quantitative data. Kaplan–Meier curves were plotted to analyze the OS, PFS, DFS rates, which was utilized to evaluate the prognostic capacity of STAMBP L1. *P* value < 0.05 was considered to be statistically significant.

### Supplementary Information

**Additional file 1**.** Fig S1**:** A**–**C**. The HCCDB database indicates that STAMBPL1 is significantly highly expressed in HCC.** D**. Single-cell sequencing data reveals that STAMBPL1 is predominantly highly expressed in HCC.** E**. The Spatial transcriptomics data demonstrates that STAMBPL1 is predominantly highly expressed in tumor cells.** Fig S2**:** A**. Investigating the binding affinity of STAMBPL1 with sorafenib using molecular docking techniques.** B**. Investigating the binding affinity of STAMBPL1 with Regorafenib using molecular docking techniques.** C**. Investigating the binding affinity of STAMBPL1 with lenvatinib using molecular docking techniques.** D**. Investigating the binding affinity of STAMBPL1 with cabozantinib using molecular docking techniques.** Fig S3**: The graphical abstract to represent overall message of our study.

**Additional file 2**:** Supplementary Table 1.** The primer sequence of STAMBPL1.** Supplementary Table 2.** The sequence information of three shRNAs targeting STAMBPL1.** Supplementary Table 3.** The detailed information of all antibodies used in this study.

## Data Availability

The datasets used in the current study are available from the corresponding author on reasonable request.

## References

[1] Brown ZJ, Tsilimigras DI, Ruff SM, Mohseni A, Kamel IR, Cloyd JM (2023). Management of hepatocellular carcinoma: a review. JAMA Surg.

[2] Kim E, Viatour P (2020). Hepatocellular carcinoma: old friends and new tricks. Exp Mol Med.

[3] Shin JY, Muniyappan S, Tran N-N, Park H, Lee SB, Lee B-H (2020). Deubiquitination reactions on the proteasome for proteasome versatility. Int J Mol Sci.

[4] Ma C, Wang D, Tian Z, Gao W, Zang Y, Qian L (2023). USP13 deubiquitinates and stabilizes cyclin D1 to promote gastric cancer cell cycle progression and cell proliferation. Oncogene.

[5] Lyu L, Lin T-C, McCarty N (2021). TRIM44 mediated p62 deubiquitination enhances DNA damage repair by increasing nuclear FLNA and 53BP1 expression. Oncogene.

[6] Liu Y, Dong C, Ren J (2023). Deubiquitination detection of p53 protein in living cells by fluorescence cross-correlation spectroscopy. ACS Omega.

[7] Zhang Q, Jiang J (2021). Regulation of hedgehog signal transduction by ubiquitination and deubiquitination. Int J Mol Sci.

[8] Hussain S, Zhang Y, Galardy P (2009). DUBs and cancer: the role of deubiquitinating enzymes as oncogenes, non-oncogenes and tumor suppressors. Cell Cycle.

[9] Nag N, Dutta S. Deubiquitination in prostate cancer progression: role of USP22. J Cancer Metastasis Treat. 2020 [cited 2024 Jan 16];2020. Available from: https://jcmtjournal.com/article/view/3504 10.20517/2394-4722.2020.23 PMC8516349 34660907

[10] Duan J, Huang D, Liu C, Lv Y, Zhang L, Chang F (2023). USP11-mediated LSH deubiquitination inhibits ferroptosis in colorectal cancer through epigenetic activation of CYP24A1. Cell Death Dis.

[11] Yan B, Guo J, Wang Z, Ning J, Wang H, Shu L (2023). The ubiquitin-specific protease 5 mediated deubiquitination of LSH links metabolic regulation of ferroptosis to hepatocellular carcinoma progression. MedComm.

[12] Wang W, Lei Y, Zhang G, Li X, Yuan J, Li T (2023). USP39 stabilizes β-catenin by deubiquitination and suppressing E3 ligase TRIM26 pre-mRNA maturation to promote HCC progression. Cell Death Dis.

[13] Zhou X, Cheng Y, Kang J, Mao G. STAM-binding protein-like 1 Promotes growth and migration of colorectal cancer by NF-κB pathway. 10.2174/0109298665272785231103104118 38008943

[14] Yu D-J, Guo C-X, Qian J, Li J, Zhu C, Jin X (2020). The long non-coding RNA NEAT1 promotes gastric cancer cell proliferation and invasion by regulating miR-103a/ STAMBPL1 axis. Technol Cancer Res Treat.

[15] Ambroise G, Yu T, Zhang B, Kacal M, Hao Y, Queiroz AL (2020). Systematic analysis reveals a functional role for STAMBPL1 in the epithelial–mesenchymal transition process across multiple carcinomas. Br J Cancer.

[16] Toh MR, Wong EYT, Wong SH, Ng AWT, Loo L-H, Chow PK-H, et al. Global epidemiology and genetics of hepatocellular carcinoma. Gastroenterology. 2023;164:766–82. 10.1053/j.gastro.2023.01.033 36738977

[17] Singal AG, Lampertico P, Nahon P (2020). Epidemiology and surveillance for hepatocellular carcinoma: new trends. J Hepatol.

[18] Marzio A, Pagano M (2020). Loss of the deubiquitinase OTULIN promotes hepatocellular carcinoma (HCC) in an mTOR-dependent manner. Cell Death Differ.

[19] Tang J, Long G, Li X, Zhou L, Zhou Y, Wu Z (2023). The deubiquitinase EIF3H promotes hepatocellular carcinoma progression by stabilizing OGT and inhibiting ferroptosis. Cell Commun Signal.

[20] Li X, Yuan J, Song C, Lei Y, Xu J, Zhang G (2021). Deubiquitinase USP39 and E3 ligase TRIM26 balance the level of ZEB1 ubiquitination and thereby determine the progression of hepatocellular carcinoma. Cell Death Differ.

[21] Zou T, Wang Y, Dong L, Che T, Zhao H, Yan X (2022). Stabilization of SETD3 by deubiquitinase USP27 enhances cell proliferation and hepatocellular carcinoma progression. Cell Mol Life Sci.

[22] Yu D, Qian J, Jin X, Li J, Guo C, Yue X. STAMBPL1 knockdown has antitumour effects on gastric cancer biological activities. Oncol Lett. 2019 [cited 2023 Jul 19]; Available from: http://www.spandidos-publications.com/10.3892/ol.2019.10789 10.3892/ol.2019.10789 PMC6781489 31611951

[23] Chen X. Targeting the deubiquitinase STAMBPL1 triggers apoptosis in prostate cancer cells by promoting XIAP degradation. 10.1016/j.canlet.2019.04.020 31004702

[24] Wang D. E3 ligase RNF167 and deubiquitinase STAMBPL1 modulate mTOR and cancer progression. 10.1016/j.molcel.2022.01.002 35114100

[25] Zhang M, Shen C, Liang H, Wu Y, Liang B (2023). Molecular cloning and function of two tumor necrosis factor receptor-associated factors genes (TRAF2 and TRAF4) from Pinctada fucata martensii. Front Mar Sci.

[26] Erba F, Di Paola L, Di Venere A, Mastrangelo E, Cossu F, Mei G (2023). Head or tail? A molecular dynamics approach to the complex structure of TNF-associated factor TRAF2. Biomol Concepts.

[27] Minicozzi V, Di Venere A, Caccuri AM, Mei G, Di Paola L (2022). One for all, all for one: the peculiar dynamics of TNF-receptor-associated factor (TRAF2) subunits. Symmetry.

[28] Tao H, Liao Y, Yan Y, He Z, Zhou J, Wang X (2021). BRCC3 promotes tumorigenesis of bladder cancer by activating the NF-κB signaling pathway through targeting TRAF2. Front Cell Dev Biol.

[29] Deen AJ, Adinolfi S, Härkönen J, Patinen T, Liu X, Laitinen T (2024). Oncogenic KEAP1 mutations activate TRAF2-NFκB signaling to prevent apoptosis in lung cancer cells. Redox Biol.

[30] Liang X, Yao J, Cui D, Zheng W, Liu Y, Lou G (2023). The TRAF2-p62 axis promotes proliferation and survival of liver cancer by activating mTORC1 pathway. Cell Death Differ.

[31] Yao J, Liang X, Xu S, Liu Y, Shui L, Li S (2024). TRAF2 inhibits senescence in hepatocellular carcinoma cells via regulating the ROMO1/ NAD+/SIRT3/SOD2 axis. Free Radic Biol Med.

[32] Wang Q, Lu W, Yin T, Lu L (2019). Calycosin suppresses TGF-β-induced epithelial-to-mesenchymal transition and migration by upregulating BATF2 to target PAI-1 via the Wnt and PI3K/Akt signaling pathways in colorectal cancer cells. J Exp Clin Cancer Res.

[33] Xu T, Rao T, Yu W-M, Ning J-Z, Yu X, Zhu S-M (2021). Upregulation of NFKBIZ affects bladder cancer progression via the PTEN/PI3K/Akt signaling pathway. Int J Mol Med.

[34] Khan S, Kwak Y-T, Peng L, Hu S, Cantarel BL, Lewis CM (2023). NLRP12 downregulates the Wnt/β-catenin pathway via interaction with STK38 to suppress colorectal cancer. J Clin Invest.

[35] Wang Z, Wu S, Wang G, Yang Z, Zhang Y, Zhu C (2023). ARHGAP21 is involved in the carcinogenic mechanism of cholangiocarcinoma: a study based on bioinformatic analyses and experimental validation. Medicina (Mex).

