# Supplementary material for: Unlocking hepatocellular carcinoma aggression: STAMBPL1-mediated TRAF2 deubiquitination activates WNT/PI3K/NF-kb signaling pathway
Source: Biol Direct. 2024 Feb 28;19:18. doi: 10.1186/s13062-024-00460-7 (PMC10903047; doi:10.1186/s13062-024-00460-7)

**Supplementary Fig 1: A-C.** The HCCDB database indicates that STAMBPL1 is significantly highly expressed in HCC. **D.** Single-cell sequencing data reveals that STAMBPL1 is predominantly highly expressed in tumor cells. **E.** The Spatial transcriptomics data demonstrates that STAMBPL1 is predominantly highly expressed in tumor cells.


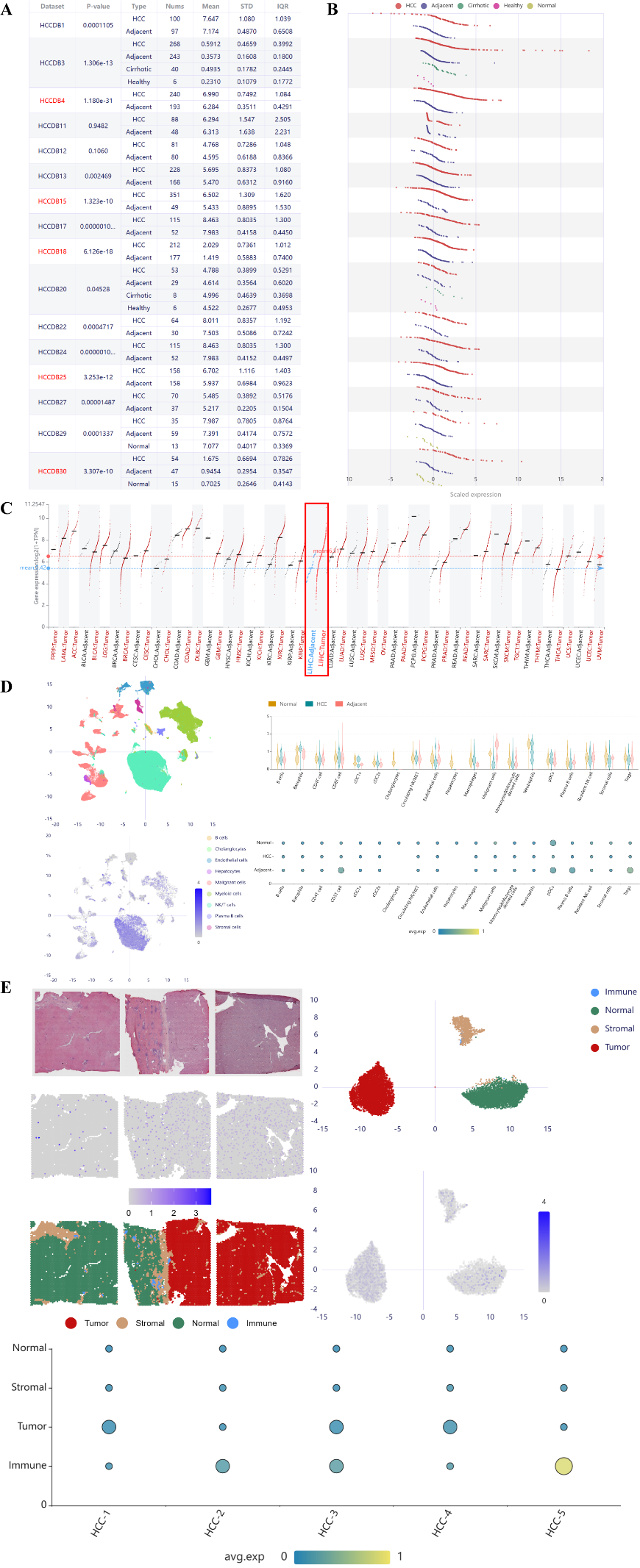


**Supplementary Fig 2: A.** Investigating the binding affinity of STAMBPL1 with sorafenib using molecular docking techniques. **B.** Investigating the binding affinity of STAMBPL1 with Regorafenib using molecular docking techniques. **C.** Investigating the binding affinity of STAMBPL1 with lenvatinib using molecular docking techniques. **D.** Investigating the binding affinity of STAMBPL1 with cabozantinib using molecular docking techniques.


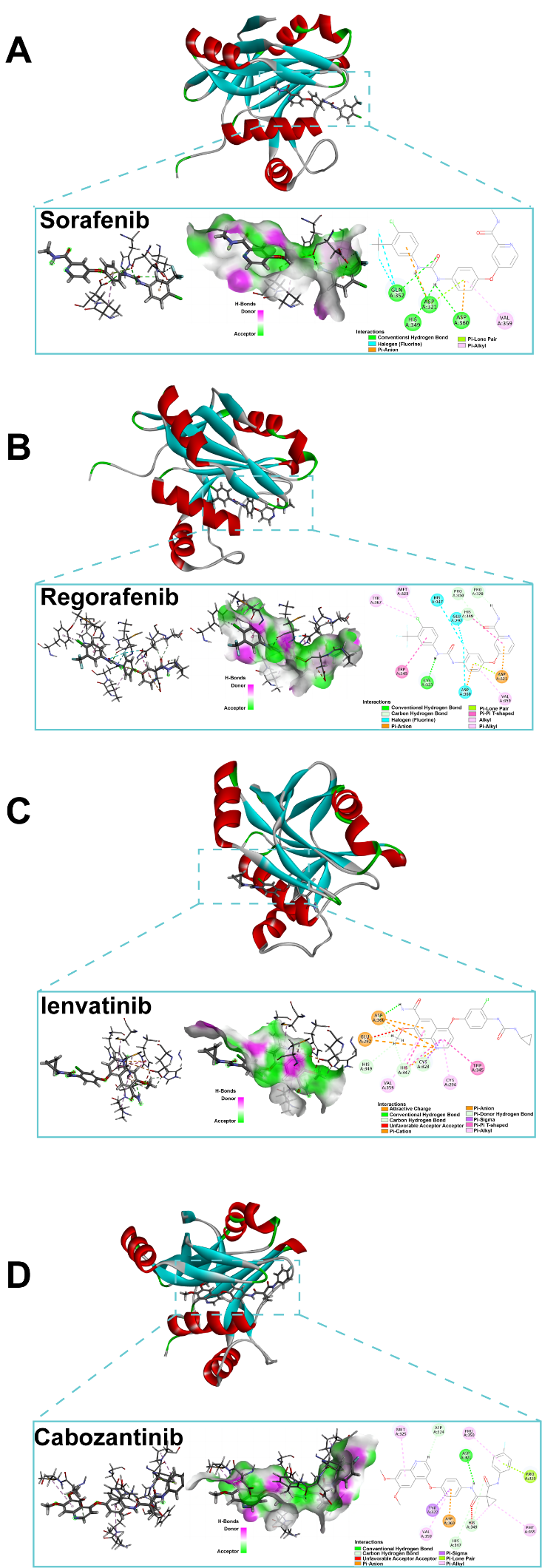


**Supplementary Fig 3:** The graphical abstract to represent overall message of our study.


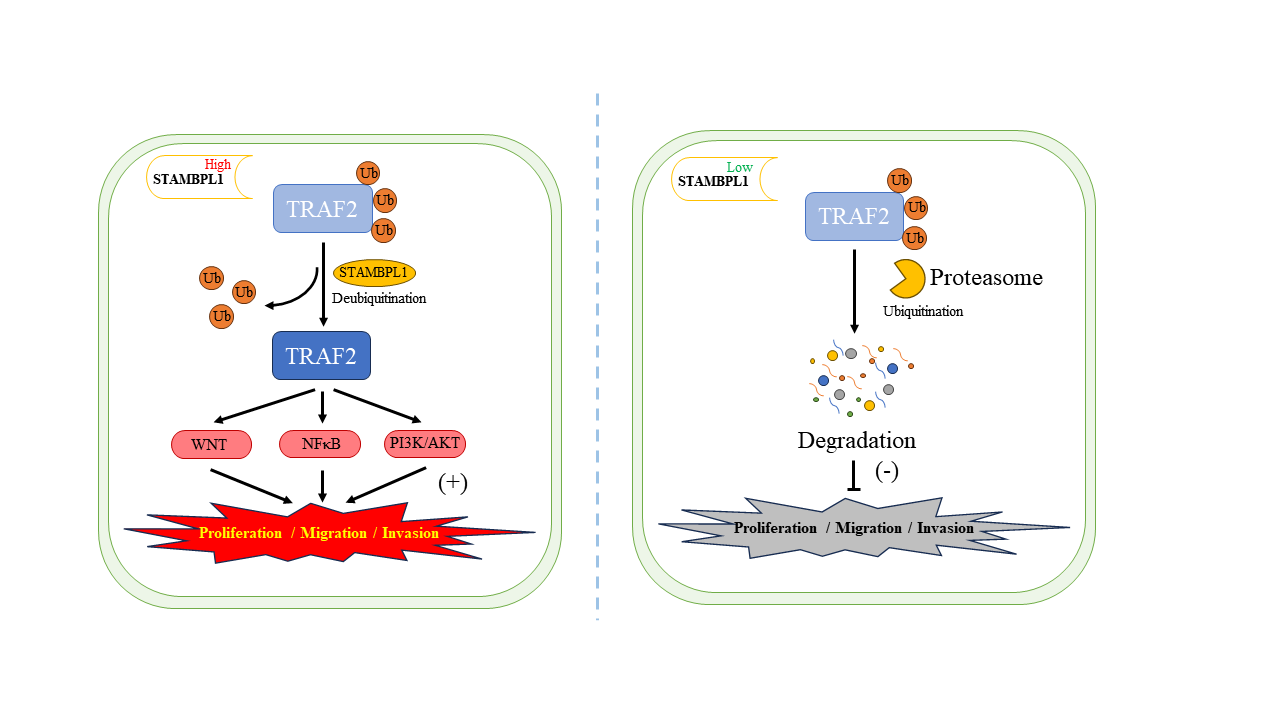

Supplement: Supplementary file 1 — Additional file 1. Fig S1: A–C. The HCCDB database indicates that STAMBPL1 is significantly highly expressed in HCC. D. Single-cell sequencing data reveals that STAMBPL1 is predominantly highly expressed in HCC. E. The Spatial transcriptomics data demonstrates that STAMBPL1 is predominantly highly expressed in tumor cells. Fig S2: A. Investigating the binding affinity of STAMBPL1 with sorafenib using molecular docking techniques. B. Investigating the binding affinity of STAMBPL1 with Regorafenib using molecular docking techniques. C. Investigating the binding affinity of STAMBPL1 with lenvatinib using molecular docking techniques. D. Investigating the binding affinity of STAMBPL1 with cabozantinib using molecular docking techniques. Fig S3: The graphical abstract to represent overall message of our study. [file 13062_2024_460_MOESM1_ESM.docx]
