# Supplementary material for: Unlocking hepatocellular carcinoma aggression: STAMBPL1-mediated TRAF2 deubiquitination activates WNT/PI3K/NF-kb signaling pathway
Source: Biol Direct. 2024 Feb 28;19:18. doi: 10.1186/s13062-024-00460-7 (PMC10903047; doi:10.1186/s13062-024-00460-7)
Supplement: Supplementary file 2 — Additional file 2: Supplementary Table 1. The primer sequence of STAMBPL1. Supplementary Table 2. The sequence information of three shRNAs targeting STAMBPL1. Supplementary Table 3. The detailed information of all antibodies used in this study. [file 13062_2024_460_MOESM2_ESM.docx]

**Supplementary Table 1:**

| Gene | Direction | Sequence |
| --- | --- | --- |
| STAMBPL1 | FORWARD | ATGTTTCCCTAAGCCCAGAAGAGC |
| STAMBPL1 | REVERSE | CACAGACGCCATCCTCTCCATC |

**Supplementary Table 2:**

| Gene | shRNA name | Sequence |
| --- | --- | --- |
| STAMBPL1 | sh-STAMBPL1_001 | GGAAGCGGATTGCTCAGAT |
| STAMBPL1 | sh-STAMBPL1_002 | CCACGACGTTACTTTAGGT |
| STAMBPL1 | sh-STAMBPL1_003 | GGACCAGACTATTGTGACA |

**Supplementary Table 3**

| Antibody | Manufacturers | Isotype | Molecular weight | Reactivity | Dilution |
| --- | --- | --- | --- | --- | --- |
| STAMBPL1 | Santa | Rabbit IgG | 50kDa | Human, Mouse | 1:1000 |
| TRAF2 | Abmart | Rabbit IgG | 55kDa | Human, Mouse | 1:1000 |
| MMP3 | Abmart | Rabbit IgG | 50kDa | Human, Mouse | 1:2000 |
| Vimentin | Abmart | Rabbit IgG | 54kDa | Human, Mouse | 1:2000 |
| E cadherin | Abmart | Rabbit IgG | 120kDa | Human, Mouse | 1:2000 |
| N cadherin | Abmart | Rabbit IgG | 140kDa | Human, Mouse | 1:2000 |
| Snail | Abmart | Rabbit IgG | 29kDa | Human, Mouse | 1:1000 |
| PI3K | Abmart | Rabbit IgG | 85kDa | Human, Mouse | 1:1000 |
| P-PI3K | Abmart | Rabbit IgG | 54kDa | Human, Mouse | 1:1000 |
| AKT | Abmart | Rabbit IgG | 56kDa | Human, Mouse，Rat | 1:1000 |
| P-AKT | Abmart | Rabbit IgG | 60kDa | Human, Mouse，Rat | 1:1000 |
| mTOR | Abmart | Rabbit IgG | 298kDa | Human, Mouse | 1:1000 |
| P-mTOR | Abmart | Rabbit IgG | 298kDa | Human, Mouse | 1:1000 |
| NF-kb | Abmart | Rabbit IgG | 65kDa | Human, Mouse | 1:5000 |
| P-NF-kb(ser529) | Abmart | Rabbit IgG | 75kDa | Human, Mouse | 1:2000 |
| P-NF-kb(ser536) | Abmart | Rabbit IgG | 65kDa | Human, Mouse | 1:2000 |
| IKK | Abmart | Rabbit IgG | 35kDa | Human, Mouse | 1:2000 |
| P-IKK (ser32316) | Abmart | Rabbit IgG | 39kDa | Human, Mouse | 1:2000 |
| APC | Abmart | Rabbit IgG | 160kDa | Human, Mouse | 1:2000 |
| OCT4 | Abmart | Rabbit IgG | 45kDa | Human, Mouse | 1:1000 |
| c-Myc | Abmart | Rabbit IgG | 57kDa | Human, Mouse | 1:2000 |
| Frizzeld-4 | Abmart | Rabbit IgG | 59kDa | Human, Mouse | 1:2000 |
| β-catenin | Abmart | Rabbit IgG | 92kDa | Human, Mouse | 1:1000 |
| LRP6 | Abmart | Rabbit IgG | 180-230kDa | Human, Mouse | 1:2000 |
| PCNA | Proteintech | Rabbit IgG | 29-36kDa | Human, Mouse | 1:1000 |
| Ki-67 | Abmart | Rabbit IgG | 358kDa | Human, Mouse，Rat | 1:1000 |
| GAPDH | Abmart | Rabbit IgG | 36kDa | Human, Mouse，Rat | 1：8000 |
| β-actin | Abmart | Rabbit IgG | 42kDa | Human, Mouse，Rat, Monkey | 1：2000 |
| CyclinD1 | Abmart |  |  |  |  |
| P21 | Abmart | Rabbit IgG | 21kDa | Human, Mouse，Rat | 1：1000 |
| GSK3 | Abmart | Mouse IgG | 46kDa | Human, Mouse， | 1：1000 |
| P-GSK3 | Abmart | Rabbit IgG | 46kDa | Human, Mouse，Rat, Monkey | 1：1000 |
| Ub | Proteintech | Rabbit IgG |  | Human, Mouse，Rat | 1：50000 |
| Flag | Abcam | Rabbit IgG |  | Human, Mouse | 1：2000 |
| Myc | Proteintech | Rabbit IgG |  | Human, Mouse，Chicken | 1：5000 |
| HA | Proteintech | Rabbit IgG |  | Recombinant Protein | 1：8000 |

**Supplementary Materials**

**Immunohistochemistry Assay**

Tissues and tumors, upon implantation, were preserved in 4% formalin and subsequently embedded in paraffin. Sections with a thickness of 4 µm underwent treatment with primary antibodies specific to STAMBPL1, TRAF2, β-catenin, c-Myc, Ki67 and PCNA (proteintech, China) during an overnight incubation at 4°C, following the blocking of endogenous peroxides and proteins. Secondary antibodies, HRP-polymer-conjugated, were applied for 1 hour at 37°C. The sections were then stained for 3 minutes in a 3,3-diaminobenzidine solution, with hematoxylin serving as a nuclei counterstain. Tumor sections were assessed in a blinded manner. Utilizing three randomly selected fields for each section, the percentage of tumors testing positive and the extent of cell staining were computed. The presented dataset reflects mean ± SEM values. Abbreviations: ns, not significant, **p* < 0.05, ***p* < 0.01, ****p* < 0.001.

**Immunofluorescence assay**

In the cell immunofluorescence experiment, target cells were seeded onto 24-well plates with coverslips at a density of 1 × 10^4 per well. Following a 24-hour incubation, wells were fixed using 4% neutral paraformaldehyde, permeabilized with 0.1% Triton X-100, and blocked with 5% BSA. Post-blocking, cells underwent overnight incubation with primary antibodies at 4°C, followed by 2 hours of incubation with secondary antibodies at room temperature. Subsequently, cells were treated with 4',6-diamidino-2-phenylindole (DAPI), and the outcomes were observed using a fluorescence inverted microscope. For the tissue immunofluorescence experiment, the tissue microarray (identical to the one used in IHC) underwent dewaxing, hydration, and antigen repair. Slides were then permeabilized with 0.2% Triton X-100 for 45 minutes. The subsequent experimental procedures mirrored those employed in cellular immunofluorescence, maintaining consistency across the methodologies.
